# VEGFC negatively regulates the growth and aggressiveness of medulloblastoma cells

**DOI:** 10.1038/s42003-020-01306-4

**Published:** 2020-10-16

**Authors:** Manon Penco-Campillo, Yannick Comoglio, Álvaro Javier Feliz Morel, Rita Hanna, Jérôme Durivault, Magalie Leloire, Bastien Mejias, Marina Pagnuzzi, Amandine Morot, Fanny Burel-Vandenbos, Matthew Selby, Daniel Williamson, Steven C. Clifford, Audrey Claren, Jérôme Doyen, Vincent Picco, Sonia Martial, Gilles Pagès

**Affiliations:** 1 Université Côte d’Azur, Institute for Research on Cancer and Ageing of Nice (IRCAN), CNRS UMR7284, INSERM U1081, Fédération Claude Lalanne (FCL), Nice, France; 2 Biomedical Department, Centre Scientifique de Monaco (CSM), Monaco, Principality of Monaco; 3 Anatomo-pathology Department, Nice University Hospital, Nice, France; 4 Wolfson Childhood Cancer Research Centre, Newcastle University, Newcastle-Upon-Tyne, UK; 5 Centre Antoine Lacassagne Cancer Institute, Nice, France

**Keywords:** Paediatric cancer, Tumour angiogenesis

## Abstract

Medulloblastoma (MB), the most common brain pediatric tumor, is a pathology composed of four molecular subgroups. Despite a multimodal treatment, 30% of the patients eventually relapse, with the fatal appearance of metastases within 5 years. The major actors of metastatic dissemination are the lymphatic vessel growth factor, VEGFC, and its receptors/co-receptors. Here, we show that VEGFC is inversely correlated to cell aggressiveness. Indeed, VEGFC decreases MB cell proliferation and migration, and their ability to form pseudo-vessel in vitro. Irradiation resistant-cells, which present high levels of VEGFC, lose the ability to migrate and to form vessel-like structures. Thus, irradiation reduces MB cell aggressiveness via a VEGFC-dependent process. Cells intrinsically or ectopically overexpressing VEGFC and irradiation-resistant cells form smaller experimental tumors in nude mice. Opposite to the common dogma, our results give strong arguments in favor of VEGFC as a negative regulator of MB growth.

## Abstract (web summary)

Manon Penco-Campillo, Yannick Comoglio et al. show that VEGFC decreases the proliferation and migration of medulloblastoma cells, as well as their ability to form pseudo vessels. Cells expressing high levels of VEGFC also form smaller tumors when subcutaneously injected into the flank of nude mice, thus highlighting a negative regulatory role for VEGFC on tumor growth.

## Introduction

Medulloblastoma (MB) is the most frequent malignant, pediatric cerebellum tumor. MB rarely occurs in adults^1^.

MB is composed of several molecular subgroups. The actual number of MB subgroups is unknown, and it is likely that each subgroup is further divided into several subtypes. The current consensus describes four principal subgroups: Wingless (Wnt), Sonic Hedgehog (SHH), Group 3 and Group 4. Wnt and SHH are characterized by aberrant activation of the corresponding signaling pathways. Since much less is known about the remaining two subgroups and no specific signaling pathway seems to play a prominent part, the consensus was to retain generic names until further discoveries about their biology. Group 3 and Group 4 MBs present *N*- and *c-myc* overexpression, p53 inactivation and deleterious chromosomal abnormalities^2–4^.

The standard of care for MB associates surgical resection of the tumor, risk-adjusted photontherapy (high-energy X-rays) and chemotherapy. This treatment leads to up to 70% survival at five years following diagnosis. Most patients suffer long-term side effects^5,6^ and relapse is fatal in all cases.

Tumor angiogenesis is related to poor prognosis, metastasis and tumor resistance to treatment^7^. However, metastasis also depends on lymphatic vessels, which are involved in draining tumor cells towards lymph nodes and beyond. Indeed, the current belief states that: (i) tumor cells produce Vascular Endothelial Growth Factor C (VEGFC), the main lymphatic endothelial cell growth factor. VEGFC induces sprouting of nearby lymphatic capillaries, intravasation of tumor cells into the neo-formed vessels, thus contributing to lymph node metastasis or even more distant tumor spreading^8–10^; (ii) tumor cells colonize lymph nodes as pre-metastatic lymphovascular niches^11^; (iii) tumor cells eventually saturate lymphatic vessels and lymph nodes, collateral lymphatic vessels with alternative lymph nodes then bypass the sentinel lymph node and participate in tumor distant metastasis^11,12^.

A lymphatic transport system has been documented in the dura mater of mammalian brain^13–16^, which allows central nervous system perfusion by the cerebrospinal fluid, drainage of the interstitial fluid towards deep cervical lymph nodes and transport of immune cells to the peripheral lymphatics^17^. Specific markers of lymphatic endothelial cells (LYVE1, VEGFR3, NRP2, PROX1, PDPN) might thus be used as prognostic markers of the severity or adverse evolution of MB.

Antiangiogenic therapies promote VEGFC-dependent lymphangiogenesis in clear cell renal cell carcinomas^18^ and radiotherapy induces a lymphangiogenic response in head and neck squamous cell carcinomas^19^. Docetaxel chemotherapy elicits VEGFR3-dependent lymphangiogenesis in breast cancer cells, thus potentiating breast tumor growth and metastasis^20^. Hence, lymphangiogenesis might constitute a common thread linking tumor aggressiveness to the reference treatment.

In the current paper, we meant to demonstrate—for the first time to our knowledge in pediatric brain tumors—the correlation between lymphatic marker expression, lymphatic vessels and MB aggressiveness; (i) in vitro, in naive or irradiated cells; (ii) in vivo, in immunodeficient xenografted mice.

We reveal that, opposite to the current dogma, VEGFC represses MB cell proliferation and migration in vitro and presents antitumoral effects in vivo.

## Results

### Correlation of lymphatic marker expression with MB subgroups

**Fig. 1:**
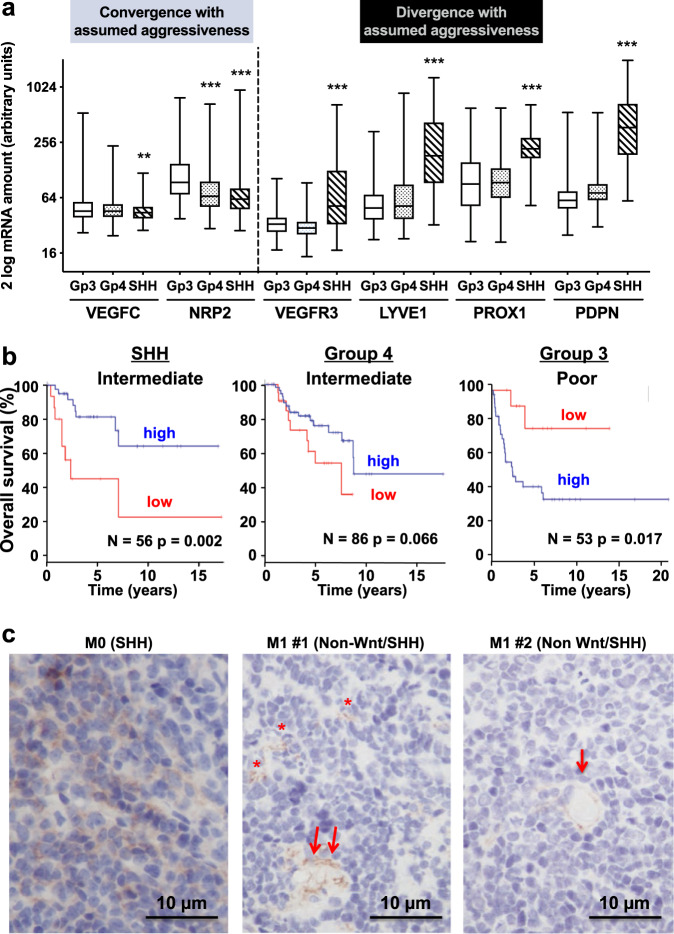
Correlation between lymphatic marker expression and MB aggressiveness. **a** The amounts of *VEGFC, NRP2, VEGFR3, LYVE1, PROX1* and *PDPN* genes were determined by analysis of the R2: Genomics Analysis and Visualization Platform (http://r2.amc.nl) data (One-way ANOVA and t-test for multiple comparison analysis; **p < 0.01; ***p < 0.001). **b** Analysis of a RNA-Seq tumor database (more than 250 patients) for overall survival of patients as a function of *LYVE1* expression and patient’s subgroup of MB. Data were binned by 25% quartile. **c** Representative images (X 400) showing the expression of podoplanin (PDPN) in patient MB tissue, as a function of metastatic status. Paraffin-embedded tumor sections were labeled with an anti-PDPN antibody. PDPN focal labeling (*) and vessel-like structures (arrows) are indicated.

We analyzed the basal expression of markers of lymphatic vessel development^21,22^ as a function of MB subgroups—in the most aggressive subgroups (SHH, Group 4, Group 3)—in the Cavalli^23^ database of the R2: Genomics Analysis and Visualization Platform (http://r2.amc.nl). Early markers of lymphatic vessel development (i.e., LYVE1, VEGFR3, PROX1) are also lymphatic endothelial cell (LEC) specification markers and VEGFC, a late marker in the development of lymphatic vessels, is rather a marker of lymphatic network expansion^22^. LYVE1, VEGFR3 and PROX1 were significantly more expressed in the SHH group (moderate to intermediate prognosis) than in more aggressive MB subgroups (groups 3 and 4): *p* < 0.001. Conversely, the SHH tumors presented a lower expression of VEGFC: *p* < 0.05 vs Group 3; *p* < 0.01 vs Group 4 (Fig. 1a). Neuropilin-2 (NRP2) is a co-receptor of VEGFR3: it enhances the VEGFC response via VEGFR3^24,25^. However, NRP2 can also function in VEGFC signaling independently of its role as a co-receptor^26,27^ and promote tumor lymphangiogenesis and metastasis. Here, in the case of MB, NRP2 mRNA levels present the same profile as VEGFC mRNA levels (Fig. 1a). This suggests that VEGFC signaling relies on NRP2 rather than and independently of VEGFR3 in MB.

To confirm this result, we analyzed the expression of VEGFC and of its receptors in the WNT subgroup and compared it to the other three subgroups (Supplementary Fig. S1a). Surprisingly, the WNT subgroup patients presented significantly higher expression of VEGFC, VEGFR2 and NRP2, when compared to the other groups, unlike VEGFR3 or CD146, which encodes a VEGFR2 co-receptor^28^. This suggests that, in MB, VEGFC signaling is mostly VEGFR2 and NRP2-directed, and that, especially in WNT, Group 4 and Group 3, VEGFR3 may not be the main receptor involved in VEGFC signaling.

Supplementary Fig. S1b presents the overall survival of WNT patients as a function of VEGFC. A low level of VEGFC tended to lead to worse outcome in the least aggressive tumors. In contrast, in the more aggressive SHH subgroup, Group 4 or Group 3 (Supplementary Fig. S1c–e), high VEGFC tended to correlate with a worse prognosis. This result underlines the dual function of VEGFC (beneficial vs. deleterious) in MB patients.

In an independent cohort of more than 250 patients^29^, high expression of LYVE1 was correlated to a longer survival in SHH group; *p* = 0.002 (Fig. 1b, left panel), while it was linked to shorter survival in Group 3; *p* = 0.017 (Fig. 1b, right panel). In Group 4, for which the clinical prognosis is intermediate, high expression of LYVE1 also tended to be favorable (Fig. 1b, middle panel).

We analyzed the R2 data considering the metastatic status of patients in each subgroup (Supplementary Fig. S2). A high amount of VEGFC mRNA was related to poor prognosis only in metastatic patients (Supplementary Fig. S2a). High levels of VEGFC mRNA were strongly associated with shorter survival metastatic patients (M1) from the SHH subgroup (Supp. Fig. S2b). Surprisingly, neither Group 4, nor Group 3 patients showed a similar result, although the trend was the same, thus emphasizing the importance of the tumor microenvironment. High expression of LYVE1 was a marker of longer survival in M0 patients (*p* = 2.5 × 10^−3^). This result was reversed in the M1 group of patients, in which high expression of LYVE1 was rather a marker of short survival (Supplementary Fig. S2c).

Finally, we studied the expression of podoplanin (PDPN), a biomarker of malignant disorders^30^, as a function of the metastatic status of MB patients. Tumors derived from M0 patients were labeled all over the epithelial cells, in a diffuse and regular manner (Fig. 1c). Contrarily, tumors derived from metastatic (M1) patients presented localized, lumen-surrounding labeling (Fig. 1c), suggesting that PDPN was expressed in lymphatic endothelial cells, only in the most aggressive MB.

These results suggest that lymphangiogenesis is associated with a poor or a favorable outcome depending on the MB genetic subgroup and metastatic status.

### Expression of lymphangiogenesis markers in MB cell lines

We mainly used two MB cell lines in our in vitro study. Daoy cells are representative of the SHH subgroup and have a well-defined epithelial morphology (Supplementary Fig. S3a). HD-MB03 cells are derived from a Group 3 tumor. HD-MB03 are semi-adherent: some of the cells develop in clusters^31^, (Supplementary Fig. S3b). This difference in morphology prompted us to study the status of our models as for epithelial vs. mesenchymal phenotype. In both cell types (Supplementary Fig. S3c), we analyzed the basal expression of three genes involved in epithelial-to-mesenchymal transition (EMT). *Cadherin-1 (CDH1)* encodes E-cadherin and *Cadherin-2 (CDH2)* encodes N-cadherin; both are cell-cell adhesion proteins. *CLDN1* encodes Claudin-1, a major constituent of the tight junction complexes that regulates the permeability of epithelia. When detected, the epithelial marker *CDH1* was low and variable. Thus, we measured *CLDN1* expression to confirm the epithelial phenotype of the cells. Daoy cells expressed 11 ± 4 times more *CDH2* mRNA, but 202 ± 25 times more *CLDN1* mRNA, than HD-MB03 cells (*n* = 3; *p* < 0.01 and *p* < 0.001, respectively). The ratio is in favor of an epithelial phenotype for Daoy cells.

Because of this cluster phenotype of HD-MB03 cells, we evaluated the expression of CD133, the main stem cell marker. HD-MB03 cells were CD133-positive while, in Daoy cells, expression was below the detection threshold (Supplementary Fig. S3d).

**Fig. 2:**
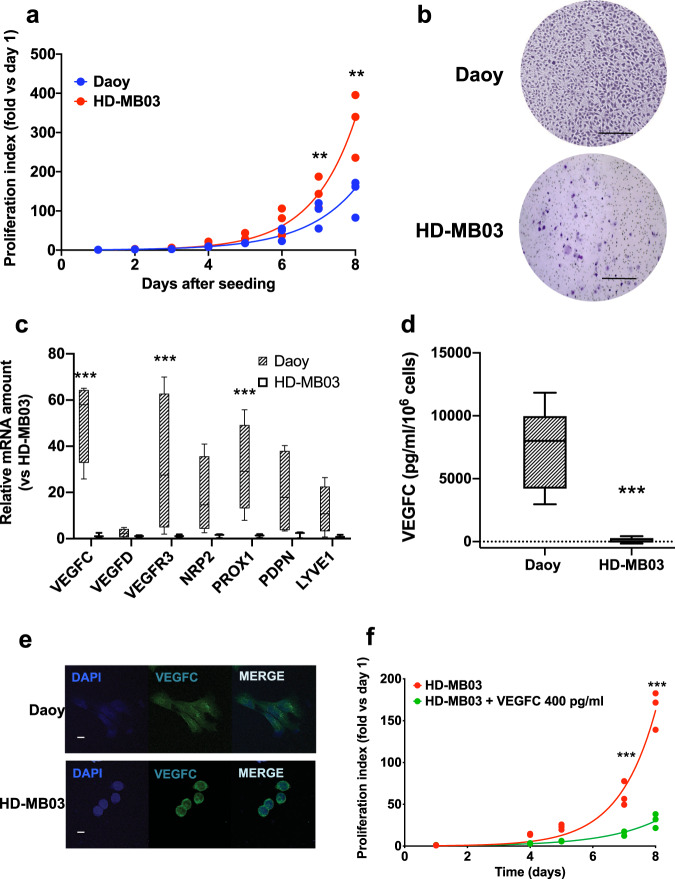
Intrinsic characteristics of MB cell lines with respect to lymphangiogenesis marker expression. **a** Average Daoy and HD-MB03 cell proliferation. Daoy or HD-MB03 cell proliferation was measured every day for eight days. *n* = 3 experiments; **p < 0.01 (Mann–Whitney analysis with Holm-Sidak correction). **b** Representative experiment of 24 h-directional migration of Daoy or HD-MB03 cells in Boyden chambers. Migrated cells were colored with Giemsa. Scale bar = 500 µm. **c** Basal relative amount of *VEGFC* mRNA and lymphangiogenic related genes in Daoy and HD-MB03 cells, as measured by RT-qPCR. *n* = 4 experiments; ***p < 0.001 (ANOVA, with multiple comparisons). **d** Basal amount of VEGFC secreted by Daoy or HD-MB03 cells. *n* = 6 experiments; *p* < 0.001. **e** Representative experiments showing the expression of VEGFC in both Daoy and HD-MB03 cells. VEGFC levels were determined by immunocytochemistry Scale bar = 10 µm. **f** Average HD-MB03 cell proliferation in the presence or absence of VEGFC in the culture medium. *n* = 3 experiments; *p* < 0.001 (ANOVA, with multiple comparisons).

We defined the in vitro behavior of these two cell lines, focusing on aggressiveness characteristics, in connection with their content in lymphangiogenesis markers. Daoy and HD-MB03 cell proliferation was consistent with their assumed aggressiveness as described by the current classification of MB subgroups^3,32^. The cell doubling time (Fig. 2a) was 1.065 day (95% CI: 0.9162 to 1.235 days) for Daoy cells. It was 0.817 day (95% CI: 0.7485 to 0.8917) for HD-MB03 cells (*p* < 0.05). To ascertain whether the slower proliferation characteristics were a general behavior of the SHH-derived tumor cells, we measured ONS-76 cell proliferation in the same conditions as above and compared it with Daoy cell proliferation (Supplementary Fig. S4a). ONS-76 cell doubling time was 0.8522 day (95% CI: 0.5875 to 1.213). It was close to the HD-MB03 doubling time and significantly different from Daoy cell proliferation (*p* < 0.05), thus showing that MB cell models present the same heterogeneity as the pathology they originate from and that any conclusion derived from these in vitro models may be drawn with caution.

Surprisingly, the highly aggressive HD-MB03 cells barely migrated in Boyden chambers, while Daoy cells had a migratory phenotype (Fig. 2b).

Since tumor growth and migration/invasion have been closely associated with VEGFC-dependent lymphangiogenesis^33–35^, we hypothesized that the above MB phenotypes were linked to the VEGFC/VEGFC-receptor axis. Daoy cell VEGFC mRNA level was 44.3 ± 4.3-fold that of HD-MB03 cells (*p* < 0.001, Fig. 2c). Moreover, VEGFC secreted protein level was 7427.0 ± 683.2 pg/ml/10^6^ cells/48 h (*n* = 11) in Daoy cells, while it was barely 133.5 ± 40.2 pg/ml/10^6^ cells/48 h (*n* = 8) in HD-MB03 cells (*p* < 0.001, Fig. 2d). Both cell types produced VEGFC protein, as shown by immunocytochemistry experiments (Fig. 2e). This result and our ELISA experiments (Fig. 2d) showed that Daoy cells secrete VEGFC, while the cytokine gets trapped in HD-MB03 cells. Although in ONS-76 cells, VEGFC mRNA level was 10 times lower than the Daoy level (Supplementary Fig. S4b), both cell lines secreted similar amounts of VEGFC (Supplementary Fig. S4c). This result indicates that VEGFC production is a common feature of Daoy and ONS-76 cells, two models of SHH MB. Moreover, it confirms that there is not a strict correlation between mRNA and protein levels, as previously described in other tumor models^36,37^.

We measured the mRNA levels of the most important lymphangiogenesis genes in MB cells. VEGFR2, VEGFR3 and NRP2, the receptors and co-receptor of VEGFC, and PROX1, the main transcription factor involved in lymphangiogenesis, were highly expressed in Daoy cells when compared to HD-MB03 cells (Fig. 2c, Supplementary Fig. S4b). In ONS-76 cells, only VEGFR2 and VEGFR3 present a high expression (Supplementary Fig. S4b), suggesting that VEGFC signaling does not use the NRP2 pathway in this cell line.

In cells representative of the Group 3 MB, the situation was more complex. HD-MB03 and D458Med cells presented low levels of VEGFC mRNA and of all the receptors and co-receptors (Fig. 2c, Supplementary Fig. S4b). However, D458Med showed a high production of VEGFC (Supplementary Fig. S4c), when compared to HD-MB03 and to D341Med, a Group 3 MB model, known for being devoid of VEGFC^38^. This result suggests that autocrine VEGFC signaling does not occur in these two models, despite their synthesis (HD-MB03) and even secretion (D458Med) of VEGFC.

Thus, VEGFC autocrine signaling is a subgroup-dependent phenomenon in MB.

Exogenous VEGFC slowed down HD-MB03 cell proliferation rate from 0.820 day (95% CI: 0.655 to 1.097) in control conditions to 1.178 day (95% CI: 0.888 to 1.75, Fig. 2f).

Thus, VEGFC and lymphatic marker levels were higher in the least aggressive cells. Unexpectedly, high VEGFC lowered two MB aggressiveness hallmarks: proliferation and migration.

### Role of VEGFC in in vitro MB cell basal aggressive phenotype

**Fig. 3:**
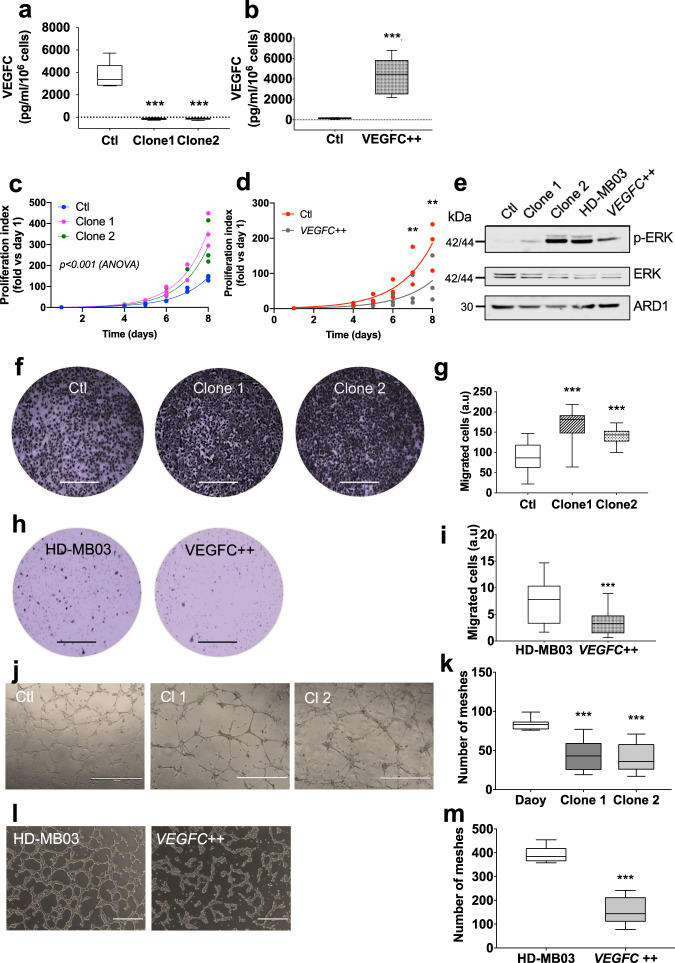
Effect of VEGFC on Daoy and HD-MB03 cell aggressiveness. **a**, **b** Assessment of the VEGFC production by VEGFC^KO^ and *VEGFC*++ cells. VEGFC levels were measured in the cell culture media by ELISA assay. Daoy: *n* = 6 experiments; HD-MB03: *n* = 9 experiments; *p* < 0.001 (ANOVA, with multiple comparisons). **c**, **d** Daoy VEGFC^KO^ and HD-MB03 *VEGFC*++ cell proliferation. **c** Daoy *VEGFC*^KO^ cell proliferation was measured over 8 days and compared to control cells. *n* = 3 experiments; ANOVA: *p* < 0.001. **d** HD-MB03 *VEGFC*++ cell proliferation was measured over 8 days and compared to wild-type cells. *n* = 3 experiments; **p < 0.01. **e** Immunoblot showing the involv**e**ment of the ERK pathway in the VEGFC-dependent cell proliferation. **f**, **g** Directional migration of Daoy *VEGFC*^KO^ cells: **f** representative experiments and **g** average results. *n* = 4 experiments**;** ***p < 0.001. **h**, **i** Directional migration of HD-MB03 cells: **h** representative experiments and **i** average results. *n* = 4 experiments; ***p < 0.001. **j**, **k** Pseudo-tube formation in Daoy *VEGFC*^KO^ cells: Representative experiments (**j**) and average results (**k**) *n* = 4 experiments; *p* < 0.001. **I**, **m** Pseudo-tube formation in HD-MB03 *VEGFC*++ cells: Representat**i**ve experiments (**I**) and average results (**m**). *n* = 4 experiments; *p* < 0.001.

To assess the role of VEGFC in Daoy and HD-MB03 cell aggressiveness, we engineered Daoy cells in which the *VEGFC* gene has been knocked-out (*VEGFC*^KO^) and a *VEGFC*-overexpressing HD-MB03 cell line (*VEGFC*++). Two independent Daoy *VEGFC*^KO^ clones were generated (Fig. 3a). On the contrary, *VEGFC* overexpression in HD-MB03 dramatically increased VEGFC secretion, then reaching the level observed in Daoy cells (Fig. 3b). We demonstrated (Fig. 3c, d) that knocking out *VEGFC* increased Daoy cell proliferation (*n* = 3; *p* < 0.001 at days 7 and 8), while *VEGFC* overexpression in HD-MB03 reduced it (*n* = 3; *p* < 0.001 at days 7 and 8). Clonogenicity ability of Daoy cells (Supplementary Fig. S3e) was increased in a significant manner (*n* = 3; *p* < 0.05 for clone 1, *p* < 0.01 for clone 2, vs. Ctl). Conversely, clonogenicity ability was significantly reduced in *VEGFC*++ HD-MB03 cells (Supplementary Fig. S3f; *n* = 3; *p* < 0.05).

We questioned the signaling pathway involved in the VEGFC-dependent proliferation. ERK pathway was activated in Daoy *VEGFC*^KO^ cells, while its activity was reduced in *VEGFC*++ HD-MB03 cells (Fig. 3e, Supplementary Fig. S3g), thus demonstrating the implication of this pathway in MB cell proliferation.

Directional cell migration was increased in Daoy *VEGFC*^KO^ cells (Fig. 3f, g) and migration was reduced in the *VEGFC*-overexpressing-HD-MB03 cells (Fig. 3h, i, *n* = 5; *p* < 0.001). Based on the vasculogenic mimicry concept^39^, we assessed the ability of our cell lines to form vessel-like structures as a function of their VEGFC content. This concept was named “lymphomimicry”. Daoy and ONS-76 cells (SHH subgroup) were both able to form pseudo-vessels (Supplementary Fig. 4d, e). Daoy: 42.3 ± 2.3 vs ONS-76: 109.4 ± 4.5 pseudo-vessels (*n* = 4). HD-MB03 cells (Group 3), which do not secrete VEGFC, were also able to form vessel-like structures (424.6 ± 18.8 (*n* = 4); Supplementary Fig. S4d, e). Conversely, D341Med and D458Med were unable to organize as pseudo-vessels, thus suggesting that the tumor cells of these two types take another metastatic route than the classic blood or lymphatic pathways.

*VEGFC* knocking-out significantly reduced the ability of Daoy cells to form vessel-like structures (Fig. 3j, k; *n* = 3; *p* < 0.001). However, HD-MB03 cells also lost their ability to organize into tubes when overexpressing VEGFC (Fig. 3l, m; *n* = 3; *p* < 0.001), thus suggesting that two mechanisms of tube formation are at stake in the two different cell lines.

We conclude that high VEGFC level is a marker of low aggressiveness in MB and that this low aggressiveness model involves an autocrine or paracrine regulation.

### Role of VEGFC in MB cell induced aggressive phenotype

**Fig. 4:**
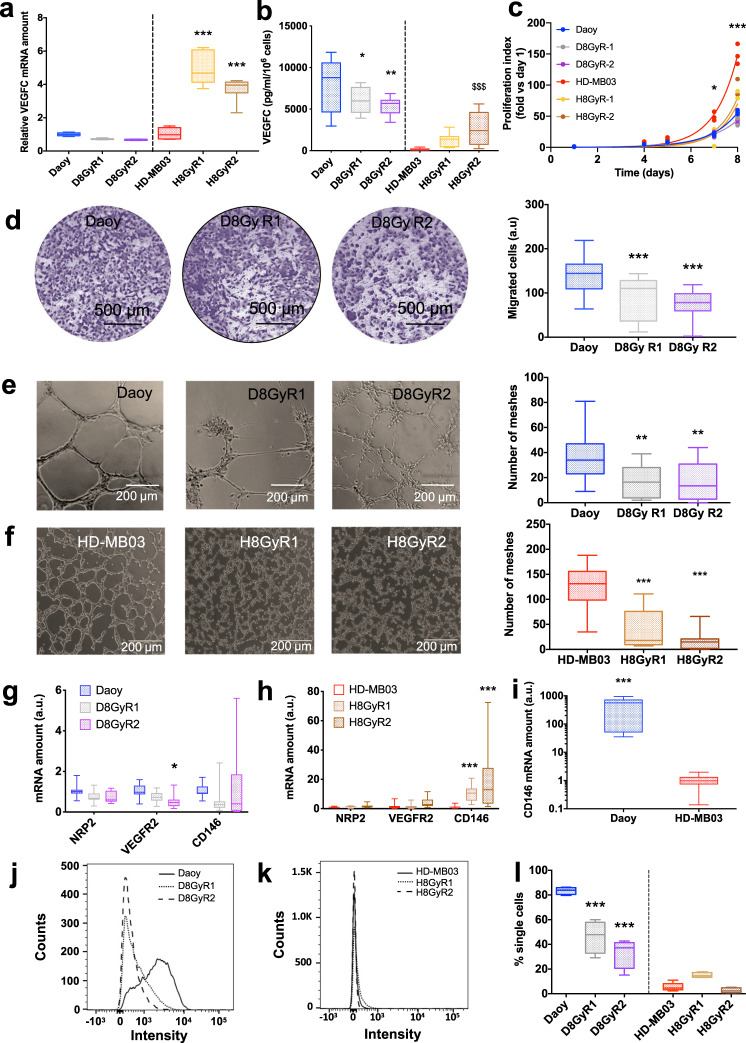
Effects of irradiation and VEGFC on Daoy and HD-MB03 cell aggressiveness. **a** VEGFC mRNA levels in Daoy or HD-MB03 irradiation-resistant cells (*n* = 6; *p* < 0.001). **b** VEGFC secretion by Daoy or HD-MB03 irradiation resistant cells (*n* = 4–6; ***p < 0.001 vs. Daoy; ^$$$^*p* < 0.001 vs. HD-MB03). **c** Proliferation of Daoy and HD-MB03 irradiation-resistant cells with respect to their own non-irradiated control cells. Cells were counted every day over 8 days. *n* = 3; ANOVA: *p* < 0.001. **d** Directional migration of Daoy irradiation**-**resistant cells: representative experiments and average results (*n* = 4; *p* < 0.001). Scale bar = 1 mm. **e**, **f** Pseudo-tube formation in Daoy or HD-MB03 irradiation**-**resistant cells: representative experiments and average results (*n* = 5). Daoy (**e**) and HD-MB03 (**f**) pseudo-tube formation was significantly altered by irradiation (**p < 0.01; ***p < 0.001, respectively). **g** qPCR expression of VEGFC receptors in Daoy irradiation-resistant cells. *n* = 6. **h** qPCR expression of VEGFC receptors in HD-MB03 irradiation-resistant cells. *n* = 6. **i** Relative expression of CD146 mRNA in Daoy and HD-MB03 cells (*n* = 6; *p* < 0.001). **j** Representative FACS measurement of CD146 membrane expression in Daoy control or irradiation resistant cells. **k** Representative FACS measurement of CD146 membrane expression in HD-MB03 control or irradiation resistant cells. **l** Average FACS measurement of CD146 membrane expression in Daoy and HD-MB03 cells (control vs irradiated). (*n* = 4; *p* < 0.001).

In order to mimic relapses following radiotherapy treatment of MB patients, we generated irradiation-resistant cells and examined their VEGFC levels. For two independent populations of Daoy cells, VEGFC expression was not altered at the mRNA level (Fig. 4a) but was significantly decreased in both populations at the protein level (Fig. 4b). However, VEGFC level stayed high, in the range of the control value. Conversely, HD-MB03 cells displayed both VEGFC mRNA and protein significant increases in both populations of irradiation-resistant cells (Fig. 4a, b). This treatment-related increase in VEGFC mRNA has previously been documented in other tumor models^18,19^. We suspect a similar upregulation process to occur in HD-MB03 cells. Daoy cell proliferation was not affected in resistant cells when compared to control cells (Fig. 4c). Conversely, both populations of irradiation-resistant HD-MB03 cells proliferated slower than control cells (Fig. 4c).

Both populations of Daoy resistant cells migrated less in Boyden chambers than their control counterparts (Fig. 4d). Irradiation-resistant HD-MB03 cells displayed no migratory phenotype, as already shown for naive HD-MB03 cells (Fig. 2). Focusing on the lymphomimicry behavior of the cells, both Daoy- and HD-MB03-resistant cells lost their ability to form tube-like structures when compared to naive cells (Fig. 4e, f). Thus, the increase in VEGFC following irradiation (Fig. 4a) was enough in HD-MB03 cells to trigger lower different features of cell aggressiveness in vitro. The high level of VEGFC in Daoy cells, whether irradiated or not, triggered the same inhibitory effect.

We measured the expression of VEGFC receptor mRNA in irradiation-resistant cells. VEGFR3 was at a low level and thus not accurately detected in any of our MB cells. In Daoy cells, VEGFC receptor mRNA did not undergo any change under irradiation, except a modest but significant (*p* < 0.05) reduction of VEGFR2 expression in only one out of two populations of irradiated cells (Fig. 4g). On the contrary, in HD-MB03 cells, CD146 mRNA was drastically increased (10 to 20-fold; *p* < 0.001), in the same proportion as VEGFC (Fig. 4h), suggesting that the irradiation-induced inhibitory effect of VEGFC is relayed, in this cell line, by the CD146 receptor in vitro. At the mRNA level, CD146 basal expression in Daoy cells was more than 500-fold the HD-MB03 level (Fig. 4i; *p* < 0.001). Strikingly, these results did not correlate with membrane expression of CD146. Indeed (Fig. 4j, l), more than 80% of Daoy cells displayed a high membrane labeling by CD146, while only 40% of Daoy irradiation-resistant cells (R1 and R2 populations) were slightly labeled by CD146 (*n* = 4; *p* < 0.001). Opposite to Daoy cells, very few (5–10%) HD-MB03 cells presented a faint membrane CD146 level, which was not modified in either irradiation-resistant population (Fig. 4k, l). This demonstrates again that two different aggressiveness mechanisms are involved in the two MB cell models.

### Mesenchymal-to-epithelial transition of irradiated MB cells

**Fig. 5:**
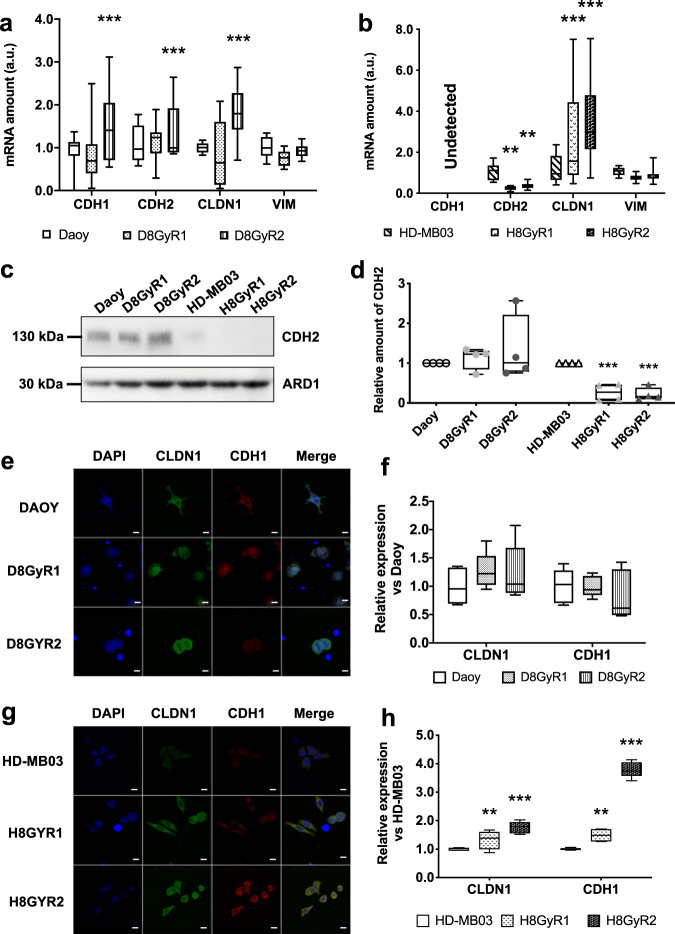
Irradiation-resistant MB cells present overexpression of epithelial markers vs. mesenchymal markers. **a**, **b** Basal relative amounts of EMT marker mRNAs (*CDH1, CDH2, CLDN1* and *VIM*) in Daoy (**a**) and HD-MB03 (**b**) irradiation-resistant cells, as measured by RT-qPCR. Only one out of two populations of Daoy resistant cells presented significantly higher levels of *CDH1* and *CDH2*, but also higher levels of *CLDN1*. HD-MB03 irradiation-resistant cells showed a very marked epithelial marker expression, correlated to a decrease in *CDH2* levels. *n* = 3 experiments; **p < 0.01; ***p < 0.001. **c** Representative immunoblot showing CDH2 expression in irradiation-resistant cells. ARD1 was used as loading control. MB cells consistently presented a decrease in CDH2 expression. **d** Average quantification of CDH2 expression in irradiation-resistant cells. *n* = 4; ***p < 0.001. **e**–**h** Immunofluorescence analysis of epithelial marker expression in Daoy (**e**, **f**) and HD-MB03 (**g**, **h**) resistant cells. CLDN1 (green) and CDH1 (red) expressions were quantified by the mean intensity of fluorescence in each field (*n* = 5 fields). Cell nucleus was labeled with DAPI. *p < 0.05; **p < 0.01; ***p < 0.001; scale bar = 10 µm.

Since cell proliferation or migration, as well as vasculogenic mimicry have been associated to the epithelial-to-mesenchymal transition (EMT) phenomenon^40,41^, we analyzed the expression of EMT genes in irradiation-resistant cells. Irradiation effect was modest if any, in Daoy cell line (Fig. 5a), since only one out of two populations of irradiation resistant Daoy cells displayed an increase in CDH1 and CLDN1 mRNAs, counterbalanced by an increase in CDH2. More neatly, both populations of HD-MB03 cells presented a significant increase in CLDN1 (*n* = 3; *p* < 0.001) coupled to a decrease in CDH2 mRNA (Fig. 5b). The protein levels were consistent with the mRNA analysis (Fig. 5c–h). Indeed, CDH2 expression was barely modified in Daoy resistant cells, while it was significantly decreased in HD-MB03 resistant cells, although expressed at very low level (Fig. 5c, d; Supplementary Fig. S4f). Immunofluorescent labeling of CDH1 and CLDN1 demonstrated a rather unchanged phenotype in Daoy cells (Fig. 5e, f) while both CDH1 and CLDN1 were induced in HD-MB03 cells (Fig. 5g, h). Hence, HD-MB03 cells adopt a more epithelial phenotype, after chronic irradiation, thus arguing against an increase in cell mobilization after irradiation.

### VEGFC effect in in vivo tumor growth

**Fig. 6:**
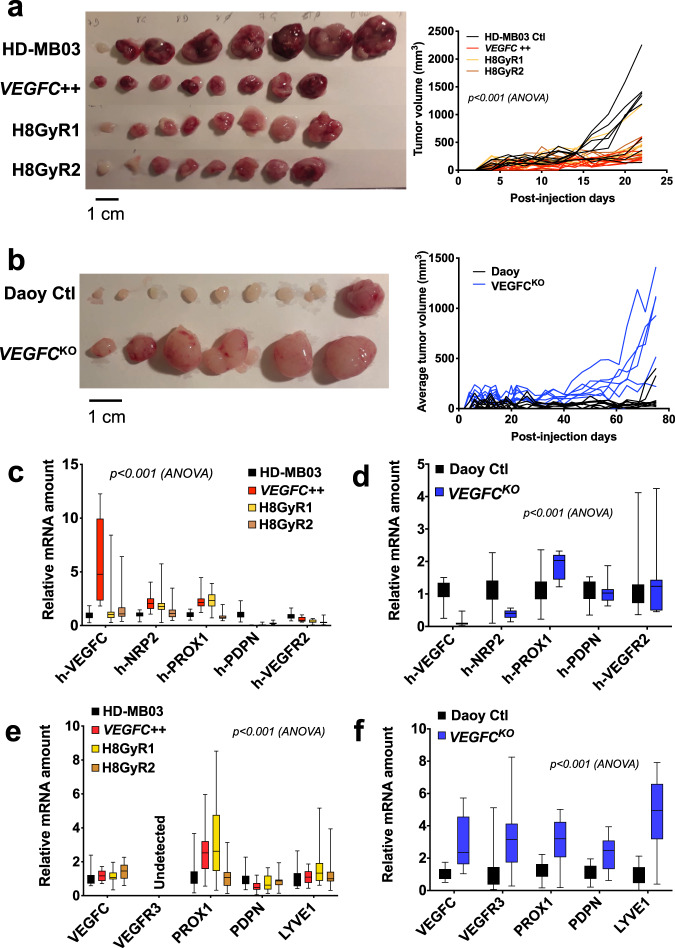
Effect of VEGFC levels on Daoy and HD-MB03 derived tumors. **a** Visual aspect and average volume of HD-MB03 cell derived tumors. **b** Visual aspect and average volume of Daoy cell derived tumors. For **a** and **b**, measurements were performed every other day. *n* = 8-9 mice per group; **a** ANOVA: *p* < 0.001; **b** *p < 0.05**;** **p < 0.01; ***p < 0.001. **c**–**f** Quantification of mRNAs involved in the lymphangiogenesis process in HD-MB03 and Daoy derived tumors, respectively (*n* = 3; *p < 0.05; **p < 0.01; ***p < 0.001). **c** Relative amount of human mRNA in HD-MB03 tumors. **d** Relative amount of human mRNA in Daoy tumors. **e** Relative amount of mouse mRNA in HD-MB03 tumors. **f** Relative amount of mous**e** mRNA in Daoy tumors.

Experimental tumors were generated by subcutaneous injection of MB cells into the flank of nude mice. This experiment was without effect on the mice health and behavior (Supplementary Fig. S5a). HD-MB03-derived tumor incidence was high: 100% of the mice were bearing tumors 4–12 days after injection in the different HD-MB03 groups (Supplementary Fig. S5b). Daoy tumor bearing mice were 90% at most (only 60% for the control group; Supplementary Fig. S5c). HD-MB03 control tumors reached 1 cm^3^ after 24 days, while 75 days were necessary to the biggest Daoy *VEGFC*^KO^ tumors to reach the same volume (Fig. 6a, b). Tumors generated from HD-MB03 cells were bigger and heavier at time of sacrifice than their *VEGFC*++ or irradiation-resistant counterparts (*n* = 8 mice/group, *p* < 0.001). There was no statistical difference between the other three HD-MB03 groups (Fig. 6a, Supplementary Fig. S5d). Daoy *VEGFC*^KO^ tumors were significantly bigger than Daoy Ctl tumors at time of sacrifice: *n* = 6–9 mice per group, *p* < 0.01 (Fig. 6b, Supplementary Fig. S5e). In the HD-MB03 groups, all the tumors looked very angiogenic, whereas Daoy tumors where much less reddish, except for *VEGFC*^KO^ (Fig. 6a, b). The in vivo tumor growth experiments were then correlated with the in vitro measurements of cell proliferation, thus confirming that the effects of VEGFC or of the absence of VEGFC may principally consist in an autocrine or paracrine effect on tumor cells.

To emphasize this conclusion, we measured gene expression in the harvested tumors. HD-MB03 tumors that contain high levels of VEGFC, also presented high levels of NRP2 (Fig. 6c). This suggests that the autocrine or paracrine positive effect of VEGFC is transmitted by this co-receptor, rather than by VEGFR3, which was not detected, nor by VEGFR2, which was not upregulated under these conditions. Surprisingly, in *VEGFC*^KO^ Daoy tumors, downregulation of VEGFC resulted in a downregulation in NRP2, while VEGFR2 level was not modified (Fig. 6d). This infers that VEGFR2, but neither by NRP2 nor VEGFR3, relayed the negative control of VEGFC on tumor.

The mouse homolog of genes involved in lymphangiogenesis was upregulated in both HD-MB03 tumors generated from engineered or irradiated cells (Fig. 6e), suggesting that lymphangiogenesis may take place around HD-MB03 VEGFC-overexpressing tumors and irradiation-resistant tumors. Likewise, *VEGFC*^KO^-derived Daoy tumors showed a 3 to 4-fold increase in the same lymphangiogenesis genes suggesting that a lymphangiogenesis process is in progress, which is consistent with the absence of neo-formed lymphatic vessels (Fig. 6f).

### Lymphangiogenic markers correlate with tumor aggressiveness

**Fig. 7:**
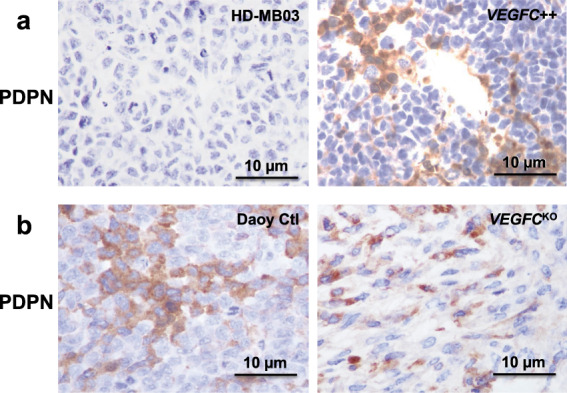
Effect of VEGFC levels on Daoy and HD-MB03 derived tumors. **a** Representative PDPN labeling of paraffin-embedded HD-MB03 tumor sections (Ctl or *VEGFC*++). **b** Representative PDPN labeling of paraffin-embedded Daoy tumor sections (Ctl or *VEGFC*^KO^).

We analyzed the correlation between the lymphangiogenic marker PDPN and tumor aggressiveness in the above-mentioned experimental tumors (Fig. 7a, b).

HD-MB03 tumors (Fig. 7a) were devoid of PDPN labeling (*n* = 8 mice). In the HD-MB03 *VEGFC*++ -injected pool of mice (Fig. 7a), only 1 out of 8 mice (12.5%) showed a strong PDPN labeling, with focal and isolated cell labeling, consistent with the high level of VEGFC in the cells.

All but one (*n* = 7) of the subcutaneous Daoy cell-derived tumors were too small (Fig. 6b) to be used in histochemistry experiments. The last one was strongly labeled (isolated cells and focal labeling) by PDPN, consistent with high levels of lymphangiogenesis markers in Daoy cells. *VEGFC*^KO^-injected mice produced bigger tumors (Fig. 6b). 4 out of 6 tumors were labeled by PDPN, with focal labeling.

## Discussion

We provide evidence supporting that the VEGFC/VEGFC receptor axes and associated lymphangiogenesis exert a beneficial effect in pediatric medulloblastoma, unlike the admitted dogma. This analysis must be taken as a pre-clinical study, using very few in vitro models of medulloblastoma, when compared to the large number of available cell lines^42^. We thus keep in mind that our conclusions should be broadened with caution.

We determined that *VEGFC* and several genes involved in lymphangiogenesis are differentially expressed in the subgroups of MB patients. The genes that are necessary for lymphatic specialization (*LYVE1, VEGFR3, PROX1*)^22^, displayed higher expression in the least aggressive subgroups, opposite to the dogma stating that lymphangiogenesis is an essential feature of several types of aggressive tumors^43–45^. Both VEGFR3^46^ and PROX1^47,48^ mRNAs are highly expressed in the developing cerebellum (https://www.proteinatlas.org/ENSG00000037280-FLT4/tissue/cerebellum), respectively in the Purkinje cells and the external granule layer, and both mRNAs regulate the neuronal development at early postnatal stages. The high level of VEGFR3 and PROX1 mRNAs might thus be related not only to lymphatic, but also to neuronal development and interestingly, specific neural progenitors from the CNS have recently been shown to promote growth and metastasis of tumors of different origin^49^.

*VEGFC*, a late gene in the lymphangiogenesis process, essential to the lymphatic system expansion^22^, presented lower expression in the patients from SHH subgroup when compared to more aggressive subgroups. Patient database analysis and immunohistochemistry experiments demonstrated that lymphatic gene expression (*VEGFC, LYVE1, PDPN*) is a function of the metastasis status. Thus, lymphangiogenic genes do not play the same role in all the MB subgroups. These genes participate in shaping the tumor cell characteristics. Especially, in SHH subgroup, *VEGFC* expression is correlated with aggressiveness, while *LYVE1, PDPN* and *PROX1* expression is inversely correlated with aggressiveness. Such discrepancies between genes and MB subgroups prompted us to study the role of lymphangiogenic genes, especially *VEGFC*, in pediatric MB cell aggressiveness.

Counter-intuitively, lymphangiogenic mRNAs (VEGFC, VEGFR3, NRP2, PROX1) were overexpressed in cells from the SHH subgroup (Daoy, ONS-76) when compared to cells from Group 3 (HD-MB03, D458Med, D341Med). Hence, the related genes are not pejorative *per se* and tumor lymphangiogenesis has beneficial effects in some instances. We demonstrate that cell irradiation, which promotes *VEGFC* and lymphangiogenesis genes, concomitantly tends to increase the epithelial phenotype of Daoy and HD-MB03 cells, thus reducing the ability of cells to disseminate^50^. Hence, as already shown in thyroid carcinoma for *PROX1*^33^, we propose that lymphangiogenesis genes have a double effect in MB. In a cancer seldom metastatic such as SHH MB, lymphangiogenic genes exert anti-tumoral effects: lymphangiogenesis paves the way for tumor cell destruction by the immune system. In a more aggressive tumor, such as Group 3 MB, in which cells grow rapidly, the higher number of cells rapidly overwhelms the immune system. Neo-lymphangiogenesis and rerouting of lymphatic vessels (synthesis of collateral lymphatic vessels) thus occurs upon vessel occlusion due to high tumor cell density^9,11^, hence participating in tumor cell propagation towards distant sites. Consistent with these hypotheses; (i) SHH cells and tumors grow slowly, thus keeping low the number of cells to get rid of by the immune system; (ii) SHH cells form pseudo-vessels in basal conditions, suggesting that transdifferentiation into lymphatic vessels is possible and give way to immune cells; (iii) knocking-out *VEGFC* gene in SHH cells results in increased aggressiveness. The opposite observations were made concerning Group 3 MB cells: although containing low amounts of lymphangiogenesis genes, they proliferate quickly and give rise to fast-growing tumors. They are able to form pseudo-vessels in basal conditions but overexpression of VEGFC reduces cell aggressiveness. We conclude that, unexpectedly, lymphangiogenesis genes, especially *VEGFC*, negatively regulate MB tumor cell aggressiveness.

Within the markers of tumor “aggressiveness”, we used the new concept of lymphangiogenic mimicry or “lymphomimicry”^51^. For the first time to our knowledge in MB, we showed that cells of three tumor models (Daoy, ONS-76 and HD-MB03) are able to organize into pseudo-vessels on matrigel. However, while Daoy—and probably ONS-76—vessel formation is highly dependent upon VEGFC, HD-MB03 cells, which do not express VEGFC, nevertheless organize into a VEGFC-free type of pseudo-vessels. We hypothesize that Daoy and ONS-76 cells form pseudo-lymphatic vessels able to hybridize with real lymphatic vessels. HD-MB03 cells transdifferentiate into pseudo-blood vessels, hence hybridizing with blood vessels and generating a different path for cell escape and metastasis. This hypothesis was partially validated by our in vivo experiments, showing that Daoy-derived tumors stay very white, with few visible blood vessels, while HD-MB03-derived tumors are extremely reddish (angiogenic, and without any PDPN labeling).

MB are usually described as angiogenic tumors^38,52^. However, as mentioned above, it is important to consider MB as a group of several pathologies inasmuch as the sub-grouping is a source of heterogeneity. Tumors derived from SHH cells show little apparent vascularization, which is part of the explanation for the small size (low infusion of O_2_ and nutriments toward the tumor) and low aggressiveness of the tumors. If any, metastasis only occurs via the lymphatic system. Conversely, tumors from Group 3 are highly vascularized. Although the vasculature is abundant, it is of poor quality^53^, thus preventing adequate doses of treatment to be provided to the tumor.

Secretion of VEGFC, expression of its receptors on tumor cells and the resulting autocrine/paracrine effect of this cytokine, are major determinants of MB tumor size. It is difficult to be categorical whether VEGFC effect is autocrine or paracrine in our models, which are multiple and for which the cells bear several types of receptors, each of them acting differently. However, tumors are not homogeneous, and most authors describe the so-called “autocrine” effect of VEGFC as the effect generated locally, on a specific type of cells—especially tumor cells—bearing the receptors, by the VEGFC secreted by this very type of cells. Paracrine effect is described as an effect occurring when VEGFC is transported by vessels (lymphatics) towards more distant targets^54–56^. To ascertain the autocrine effect of VEGFC, internalization of tagged-VEGFC receptors might be implemented. However, it is probable that this effect might be compensated by the other types of receptors (NRP2, CD146).

In two different models of MB, high amounts of VEGFC/VEGFC receptors correlate with lower tumor size and lower migration of tumor cells. Our hypothesis is that the initial effect of VEGFC is to reduce MB cell proliferation/migration, thus keeping the tumor in a state where it can more easily be attacked and destroyed by the immune cells. Later on, when this system gets saturated, or for a fast-growing tumor such as Group 3 tumors, overwhelming the system leads to synthesis of more lymphatic vessels and to metastasis.

Thus, an efficient MB treatment must take into account: (i) the differences between subgroups and (ii) the time of administration.

Daoy cells have an epithelial phenotype^31^, while HD-MB03 cells are more mesenchymal. Moreover, HD-MB03 are CD133^+^ cells^57^, which is associated with increased metastasis and poor outcome of patients^57,58^. In vivo, HD-MB03 cells gave rise to rapid-growing tumors. This high rate of tumor growth was reduced by *VEGFC* expression by the cells. The same result was achieved in vitro, by exogenous addition of VEGFC to the HD-MB03 cells. This observation was surprising. It is possible to consider VEGFC as a treatment for Group 3 MB, under certain conditions. This provocative suggestion needs to be reinforced by more in vivo experiments in immunocompetent mice, but this is the first step approaching a potential treatment for the aggressive Group 3 MB.

Our results seem inconsistent with gene expression in patients. This demonstrates the complexity of a whole tumor, the role of the tumor microenvironment, blood, lymphatic and immune systems and of phenomena such as hypoxia, which are not totally understood yet^59–61^. The effect of tumor microenvironment has previously been demonstrated in the lab. Indeed, *VEGFC* knock-out has opposite effects on tumor growth in immunodeficient or immunocompetent mice^37^.

In conclusion, unexpectedly, the common thread between the different MB subgroups is the downregulation of tumor cell aggressiveness by VEGFC. We infer that this phenomenon is involved in the early regulation of tumor development by the immune system, both by maintaining tumors at a small size and by generating the tumor lymphatic vessels conveying these small tumors towards lymph nodes, where they are degraded. These observations pave the path for the development of new therapeutic strategies based on combined treatment between VEGFC and immune checkpoint inhibitors, in MB patients.

## Materials and methods

### Experimental models

*MB cells*: Human medulloblastoma cell line Daoy (HTB-186) was purchased from ATCC and maintained in MEMα with Glutamax (Thermo Fisher, Montigny-le-Bretonneux, France) supplemented with 10% fetal bovine serum (FBS; Dutscher, Brumath, FRANCE) and 1 mM sodium pyruvate. ONS-76 cell line was a kind gift of Dr. Celio Pouponnot (Institut Curie, Paris, FRANCE). They were maintained in the same medium as Daoy cells. HD-MB03 cell line was purchased from DSMZ (Leibniz, Germany) and maintained in MEMα with Glutamax supplemented with 10% FBS and 1 mM sodium pyruvate. D458Med cells were a kind gift of Dr. Celio Pouponnot (Institut Curie, Paris, FRANCE). They were maintained in Improved MEM (Thermo Fisher) supplemented with 10% FBS. D341Med cell line (HTB-187) was purchased from ATCC and maintained in MEMα with Glutamax supplemented with 20% FBS.

*VEGFC*^*KO*^ *clones*: The *VEGFC* gene was knocked-out in wild type Daoy cells (WT-Daoy) by the CRISPR-Cas9 technique^62^. Briefly, a human *VEGFC* target oligonucleotide (5′-GAGTCATGAGTTCATCTACAC-3′) was cloned into the pX330-U6-Chimeric_BB-CBh-hSpCas9 vector (gift of Dr. Feng Zhang; Addgene plasmid # 42230). Two *VEGFC*^KO^ clones were obtained by PEI transfection (Tebu Bio, Le-Perray-en-Yvelines, FRANCE) of the resulting vector into Daoy cells and further selection on 5 µg/ml puromycin (InvivoGen, Toulouse, France), for 10–15 days. Control cells (Ctl) were obtained by transfection of WT-Daoy cells by an empty pX330 vector and puromycin selection. The mutations leading to *VEGFC* invalidation were revealed, for each clone, by genomic DNA sequencing, using the following primers: Sense, 5′-TTGTGTTAGGGAACGGAGCAT-3′; Antisense, 5′-AGAACCAGGCTGGCAACTTC-3′. Clone 1 was homozygous while clone 2 was heterozygous, but in both cases, no VEGFC protein was translated (Supp. Table S2). Effectiveness of *VEGFC* invalidation was confirmed by ELISA assay of VEGFC production.

*VEGFC++ cells*: HD-MB03 cells that overexpress *VEGFC* were generated by lentiviral infection. *VEGFC* was first amplified by PCR from the pQCXIP-VEGFC retroviral vector, using the following primers: sense, VEGFC_SpeI_For: 5′-TTTACTAGTATGCACTTGCTGGGCTTC-3′; antisense, VEGFC_XhoI_Rev: 5′-TTTCTCGAGTTAGCTCATTTGTGGTCTTTTCC-3′. pQCXIP-VEGFC was a gift of Dr. Michael Grusch; Addgene plasmid # 73012). The amplified fragment was purified, SpeI / XhoI-digested and inserted into the pLenti6.3/V5–DEST™ plasmid, from Invitrogen (Thermo Fisher). WT-HD-MB03 cells were infected according to the protocol available at www.addgene.com. A total population of cells was generated by selection with 10 µg/mL blasticidin for 10–15 days. The cell line was named *VEGFC*++ . Control HD-MB03 cells (Ctl) were obtained by WT-HD-MB03 infection with the pLenti6.3/V5–GW/lacZ plasmid.

*X-ray resistant cells*: Highly confluent Daoy and HD-MB03 cells (two populations each) were X-ray irradiated every week for 10 weeks with no cell sub-culturing, using a Faxitron cabinet X-ray irradiator (160kV-6.3 mA; Edimex, Le Plessis-Grammoire, FRANCE). An 8-Gy dose was delivered at each irradiation. After 10 weeks, naive and resistant cells were irradiated once at 8 Gy and cell viability was assessed 5 days later using the ADAM cell counter (MBI, Dorval, CANADA). Viability was close to 80% for both Daoy and HD-MB03 resistant cells, while naive Daoy cells survived at 60% after one such irradiation and naive HD-MB03 at only 40% (Supp. Fig. S6).

All cells were cultured at 37 °C in a humidified atmosphere with 5% CO_2_. For all experiments, cell lines were maintained for no more than 2 months.

### Antibodies

Cell Signaling Technologies (CST) anti-CD133, CD146, CDH2, CLDN1, ERK were purchased from Ozyme (Saint-Quentin-en-Yvelines, FRANCE). Anti-p-ERK and anti-PDPN were purchased from Abcam (Paris, FRANCE), as well as goat anti-rabbit Alexa 488 and goat anti-mouse Alexa 594 IgGs. Invitrogen anti-α-tubulin was purchased from Thermo Fisher. Anti-ARD1 was a homemade polyclonal antibody made against the last 20 C-terminal amino acids of the human ARD1 sequence, as described earlier^63^. HRP-coupled anti-rabbit and anti-mouse IgG (Promega, Charbonnières-les-Bains, FRANCE) were used (1:5000) as secondary antibodies for immunoblotting experiments.

A list of all the antibodies used in this study is provided in Supplementary Table S3.

### Chemicals

All standard chemicals were purchased from Sigma-Aldrich, unless otherwise stated.

### Cell proliferation

1500 Daoy or ONS-76 cells or 7500 HD-MB03 cells were seeded in six-well plates in triplicates and cells were counted every day for 8 days, using a Coulter counter (Villepinte, FRANCE). The relative number of cells (vs day 1) was assessed daily. Cell growth was fitted to an exponential growth equation:

$$Y = Y0 * exp(k * X)$$

with *Y* = Cell number at day X;

*Y*0 = Cell number at day 0 (graphically calculated);

*X* = Day following cell plating (cells plated at *X*0 = 0);

*k* = Exponential growth constant rate.

Doubling times were compared.

### Clonogenicity assay

In order to test the VEGFC-dependent clonogenic ability, *VEGFC*^WT^ and *VEGFC*^KO^ cells (Clones 1 and 2) were seeded in triplicates in 6-well plates (150 cells/well). The clones were fixed 9 days later with ice-cold ethanol and colored with Giemsa. We set the cut-off at clones bigger than 50 cells and calculated the plating efficiency (PE) as:

$${\mathrm{PE}}({\mathrm{\% }}) = ({\mathrm{number}}\,{\mathrm{of}}\,{\mathrm{colonies}}\,{\mathrm{formed}})/({\mathrm{number}}\,{\mathrm{of}}\,{\mathrm{seeded}}\,{\mathrm{cells}}) * 100$$

### Cell migration

Daoy or HD-MB03 cells were deprived of serum overnight. 25,000 Daoy or 50,000 HD-MB03 cells were plated in serum-free medium into the upper compartment of pre-wetted inserts (24-well plates fit, translucent PET membrane, 8.0 µm pore size, Falcon, Thermo Fisher). 10% serum-medium was added to the lower chamber. After 20 h incubation at 37 °C, in a humidified atmosphere with 5% CO_2_, migrated cells were fixed with 3% paraformaldehyde and stained with Crystal Violet. Bright field images were taken with a 10× objective Evos XL Core Cell Imaging system (Thermo Fisher). The cells were counted by the ImageJ (NIH) software.

### RNA isolation for reverse transcription and real-time PCR

Cells were seeded in six-well plates (150,000 cells/well, Daoy or ONS-76 cells; 200,000 cells/well, HD-MB03 or D458 cells) and grown for 48 h, at 37 °C, in a humidified atmosphere with 5 % CO_2_. RNA was extracted with the RNeasy Mini kit (Qiagen, Courtabœuf, FRANCE), according to the manufacturer’s recommendations. Reverse transcription was performed using the QuantiTect® Reverse Transcription Kit (Qiagen) according to the manufacturer’s instructions. Real-time PCR reactions were carried out in triplicates, on the StepOne Plus Real-time PCR system (Thermo Fisher), using the Takyon™ ROX SYBR® 2X MasterMix dTTP Blue (Eurogentec, Angers, FRANCE) and with the following parameters: one cycle at 95 °C for 20 s followed by 40 amplification cycles at 95 °C for 3 s and 60 °C for 30 s, and finally a dissociation step at 95 °C for 15 s, 60 °C for 1 min, 95 °C for 15 s. Specific primers (Supplementary Table S2) were synthesized by Sigma-Aldrich (St. Quentin Fallavier, FRANCE). The housekeeping Ribosomal Protein Lateral Stalk Subunit P0 gene (RPLP0, 36B4, 60 S ribosomal subunit protein) was used for data normalization. Data were analyzed as comparative Ct (ΔΔCt; relative quantitation).

### Protein extraction and immunoblot assays

Daoy and HD-MB03 cells were cultured to sub-confluence, then lysed on ice with 1X Laemmli buffer (6 mM Tris-Cl pH 6.8, 2% SDS, 10% glycerol, 5% β-mercaptoethanol). The lysates were sonicated for 30 s and protein amount was determined by the Pierce™ BCA Protein Assay Kit assay (Thermo Fisher). Sample proteins were reduced by heating at 95 °C for 5 min with 5% β-mercaptoethanol. 50 μg of proteins were loaded on 10% polyacrylamide gels and SDS-PAGE was performed using the Mini-Protean® Tetra Vertical Electrophoresis Cell (Biorad, Marnes-la-Coquette, FRANCE). The proteins were transferred onto PVDF membranes in Tris-Glycine buffer (25 mM Tris, 192 mM glycine, pH 8.3) + 20% ethanol (v:v) using the Hoefer wet blotting system (Thermo Fisher). Membranes were air-dried and blocked with 3% BSA, at room temperature for 1 h, then immunoblotted overnight with primary antibodies diluted in 3% BSA, at 4 °C. Membranes were washed with water, incubated with HRP-conjugated secondary antibodies, at room temperature, for 1 h. After final washing with water, the Advansta WesternBright Quantum HRP substrate (Diagomics, Blagnac, FRANCE) was used as detection reagent.

### Flow cytometry

Wild type or irradiation resistant Daoy or HD-MB03 cells (10^6^ cells) were softly dissociated with 1X accutase (HyClone HyQTase, Fisher Scientific). The cells were then labeled with a mouse monoclonal PE-conjugated anti-CD146, (BioCytex, Marseille, FRANCE). Fluorescence was measured using the 488 and 633 nm lasers of a flow cytometer (FACS Canto - BD Biosciences, FRANCE). The gating strategy of these experiments is described in Supplementary Fig. S7. The data were collected with the BD FACSDiva software application. Percentages of CD146-labeled isolated cells were calculated with FlowJo® software version 18.0 (Flexera, Hamburg, GERMANY).

### Immunocytochemistry

Immunofluorescence studies were performed on medulloblastoma cell lines. Cells were plated (10^5^ Daoy, 1.5*10^5^ HD-MB03) into 12-well plates with 14 mm coverslips first coat with 0.1 mg/ml poly-l-Lysine (Sigma-Aldrich). After 24 h the cells were fixed in 4% PFA, 25 min. After two PBS washing, cells were stained 1 h at room temperature with primary antibody (1:100 dilution) in blocking solution (3% BSA, 0.1% saponine, PBS). After washing, cells were incubated with fluorochrome-conjugated (AlexaFluor 488, and AlexaFluor 594) secondary antibodies for 1 h at room temperature. Nuclei were visualized with 1 μg/ml DAPI (4, 6-diamidino-2-phenylindole) (Sigma-Aldrich). Images were acquired with a 63× (1.4 numerical aperture) oil immersion objective and Zeiss LSM 870 confocal laser-scanning microscope. Images were processed using FIJI software.

### Enzyme-linked immunosorbent assay (ELISA)

Cells were seeded in 12-well plates in triplicates (50,000 cells/well for Daoy or ONS-76 cells, 100,000 cells/well for HD-MB03, D458Med or D341Med). The cells were grown for 48 h, at 37 °C, in a humidified atmosphere with 5% CO_2_. The supernatants were then recovered, and the cells of each well were counted for data normalization. VEGFC was quantified from supernatants with the R&D Systems Human VEGFC Duoset ELISA kit (Minneapolis, MN, USA), according to the manufacturer’s recommendations. Data are presented as secreted VEGFC in pg/ml/10^6^ cells/48 h.

### Pseudo-vessel formation assay

Cells were deprived of serum overnight. They were then seeded (75,000 Daoy cells, 200,000 ONS-76 cells or 300,000 HD-MB03, D458Med or D341Med cells per well) in 24-well plates coated with Corning® Matrigel® Matrix, hESC-Qualified (VWR, Fontenay-sous-Bois, FRANCE). The plates were then incubated at 37 °C, 5% CO_2_ for 6–30 h. Representative pictures were taken with the Evos XL Core Cell Imaging system at low magnification (4X).

### Subcutaneous xenografts

This study was conducted in compliance with the National Charter on the ethics of animal experimentation. Our experiments were approved by the “Comité National Institutionnel d'Éthique pour l’Animal de Laboratoire (CIEPAL)” (reference: NCE/2017–383). 10^6^ Daoy cells or 0.5 × 10^6^ HD-MB03 cells were injected, in medium containing 50% Corning® matrigel® matrix (VWR), subcutaneously, into the flank of 6-week-old NMRI-Foxn1nu/Foxn1nu female mice (Janvier Labs, Le Genest-Saint-Isle, France). Tumor volume was measured every other day with a caliper and calculated as follows:

$$V = \pi /6 \times L \times l^2$$

where *L* = tumor length; *l* = tumor width (in a 2D space, tangential to the mouse skin).

The experiment was stopped when the tumors of one experimental group reached a volume of 1000 mm^3^.

### Immunohistochemistry and immunofluorescence

Each tumor was fixed in 10% formalin and paraffin embedded. Morphologic examination was performed upon Hematoxylin and Eosin stained sections (3–4 μm).

Podoplanin immunohistochemistry was performed on paraffin-embedded tumor sections using a mouse antibody against podoplanin (clone D2-40, Diagomics, Blagnac, FRANCE; 1:25). Deparaffinization, rehydration and antigen retrieval were performed using the pretreatment PT Link device (Dako, Agilent, Les Ulis, FRANCE), at pH 9. Primary antibody was incubated for 20 min. Revelation was performed using the Dako EnVision^TM^ Flex/HRP revelation kit, with diaminobenzidin as chromogen. Sections were counterstained with haematoxylin.

Five micrometers thick frozen serial sections were fixed 30 min in 4% PFA before immunostaining procedure. Prior to primary antibody application, tissue sections were blocked 60 min in phosphate-buffered saline (PBS) containing 3% bovine serum albumin, 5% horse serum, 5% goat serum and 1% bovine serum albumin (Sigma-Aldrich). Incubation with primary antibodies was carried out overnight at +4 °C. Negative controls were left with blocking solution, without primary antibody overnight at +4 °C. Incubation with fluorichrome-conjugated (AlexaFluor 488 and AlexaFluor 594) secondary antibodies, which were specific to each primary antibody, was performed for 60 min at room temperature in dark. DAPI was used to visualize nuclei.

### Patient sample immunohistochemistry

In collaboration with the Nice (Dr. Fanny Burel-Vandenbos) and Marseille (Dr. Nicolas André) hospitals, we analyzed sections from formalin-fixed and paraffin-embedded MB samples for lymphatic marker labeling, as described elsewhere^19^. Briefly, the samples were incubated at room temperature with monoclonal, primary mouse anti-human PDPN and CD31 antibodies and biotinylated secondary antibodies. Labeling was detected with the diaminobenzidine substrate against a hematoxylin counterstain. An accredited clinical pathologist (Dr. Burel-Vandenbos) evaluated marker expression.

### Technical resources

Dr. Steven C. Clifford (Newcastle-upon-Tyne, UK) provided us with a databank of 250 samples of MB, with transcriptomic and clinical data, including overall and progression-free survival^29^. We also compiled and analyzed MB data from the “R2: Genomics Analysis and Visualization Platform” (http://r2.amc.nl) for lymphangiogenesis gene expression.

### Statistics and reproducibility

Results were expressed in the text as mean ± SEM of at least three independent experiments. Unless otherwise stated, statistical analyses were performed using one-way or two-way ANOVA tests with Dunnett’s multiple comparison tests. For two independent groups, Mann-Whitney analyses were performed. Results were considered significant when *p*-value <0.05.

For in vitro experiments, 3 to 5 samples were used to assess a biological effect. Most authors consider that replicating three times the same experiment, taking care that cell culture conditions do not change is enough to accurately describe a biological phenomenon. We increased the number of samples for qPCR experiments, where variability is higher. For in vivo experiments, each group of animals is composed of 8–10 individuals. Our experiments did not require blind tests.

No data were excluded.

### Reporting summary

Further information on research design is available in the Nature Research Reporting Summary linked to this article.

## Supplementary information

Supplementary Information

Reporting Summary

## Data Availability

The datasets generated during and/or analyzed during the current study are available in the Figshare repository, https://figshare.com/articles/dataset/CommsBio_19-1661A-Raw_data_xlsx/12881351.
