# Author Correction: VEGFC negatively regulates the growth and aggressiveness of medulloblastoma cells

**DOI:** 10.1038/s42003-020-01502-2

**Published:** 2020-12-07

**Authors:** Manon Penco-Campillo, Yannick Comoglio, Álvaro Javier Feliz Morel, Rita Hanna, Jérôme Durivault, Magalie Leloire, Bastien Mejias, Marina Pagnuzzi, Amandine Morot, Fanny Burel-Vandenbos, Matthew Selby, Daniel Williamson, Steven C. Clifford, Audrey Claren, Jérôme Doyen, Vincent Picco, Sonia Martial, Gilles Pagès

**Affiliations:** 1 Université Côte d’Azur, Institute for Research on Cancer and Ageing of Nice (IRCAN), CNRS UMR7284, INSERM U1081, Fédération Claude Lalanne (FCL), Nice, France; 2 Biomedical Department, Centre Scientifique de Monaco (CSM), Monaco, Principality of Monaco; 3 Anatomo-pathology Department, Nice University Hospital, Nice, France; 4 Wolfson Childhood Cancer Research Centre, Newcastle University, Newcastle-Upon-Tyne, UK; 5 Centre Antoine Lacassagne Cancer Institute, Nice, France

**Keywords:** Paediatric cancer, Tumour angiogenesis

Correction to: *Communications Biology* 10.1038/s42003-020-01306-4, published online 16 October 2020.

In the original published version of the Article, the legend of Fig. 3 contained an error in which a scale bar definition was missing in the description for panel L. In the legend for Fig. 4, there was an incorrect scale bar definition in the description for panel D, which was originally ‘scale bar = 1 mm’. In addition, the title of Supplementary Fig. S4 has been corrected from “Follow-up of MB cell xenograft into Nude mice” to “MB group-related features of MB cells” to accurately describe the presented data.

These errors have been corrected in both the PDF and HTML versions of the article and the HTML has been updated to include a corrected version of the Supplementary information, which is additionally attached to this Correction.

## Supplementary information

Supplementary items

