# Supplementary material for: VEGFC negatively regulates the growth and aggressiveness of medulloblastoma cells
Source: Commun Biol. 2020 Oct 16;3:579. doi: 10.1038/s42003-020-01306-4 (PMC7568583; doi:10.1038/s42003-020-01306-4)
Supplement: Supplementary file 2 — Reporting Summary [file 42003_2020_1306_MOESM2_ESM.pdf]

nature research

Corresponding author(s): Sonia MARTIAL

Last updated by author(s): Sep 11, 2020

Reporting Summary

Nature Research wishes to improve the reproducibility of the work that we publish. This form provides structure for consistency and transparency in reporting. For further information on Nature Research policies, see Authors & Referees and the Editorial Policy Checklist .

Statistics

For all statistical analyses, confirm that the following items are present in the figure legend, table legend, main text, or Methods section.

n/a	Confirmed	
☐	☒	The exact sample size (n) for each experimental group/condition, given as a discrete number and unit of measurement
☐	☒	A statement on whether measurements were taken from distinct samples or whether the same sample was measured repeatedly
☐	☒	The statistical test(s) used AND whether they are one- or two-sided<br>*Only common tests should be described solely by name; describe more complex techniques in the Methods section.*
☒	☐	A description of all covariates tested
☒	☐	A description of any assumptions or corrections, such as tests of normality and adjustment for multiple comparisons
☐	☒	A full description of the statistical parameters including central tendency (e.g. means) or other basic estimates (e.g. regression coefficient) AND variation (e.g. standard deviation) or associated estimates of uncertainty (e.g. confidence intervals)
☐	☒	For null hypothesis testing, the test statistic (e.g. F, t, r) with confidence intervals, effect sizes, degrees of freedom and P value noted<br>*Give P values as exact values whenever suitable.*
☒	☐	For Bayesian analysis, information on the choice of priors and Markov chain Monte Carlo settings
☒	☐	For hierarchical and complex designs, identification of the appropriate level for tests and full reporting of outcomes
☒	☐	Estimates of effect sizes (e.g. Cohen's d, Pearson's r), indicating how they were calculated

Our web collection on statistics for biologists contains articles on many of the points above.

Software and code

Policy information about availability of computer code

Data collection	None
Data analysis	None

For manuscripts utilizing custom algorithms or software that are central to the research but not yet described in published literature, software must be made available to editors/reviewers. We strongly encourage code deposition in a community repository (e.g. GitHub). See the Nature Research guidelines for submitting code & software for further information.

Data

Policy information about availability of data

All manuscripts must include a data availability statement. This statement should provide the following information, where applicable:

- Accession codes, unique identifiers, or web links for publicly available datasets
- A list of figures that have associated raw data
- A description of any restrictions on data availability

All the data provided in this paper have been made public and accessible in the FigShare public repository as requested by the Nature policy on availability of data. The data is accessible at the following URL: https://figshare.com/articles/dataset/CommsBio_19-1661A-Raw_data_xlsx/12881351

Field-specific reporting

Please select the one below that is the best fit for your research. If you are not sure, read the appropriate sections before making your selection.

☒ Life sciences ☐ Behavioural & social sciences ☐ Ecological, evolutionary & environmental sciences

Life sciences study design

All studies must disclose on these points even when the disclosure is negative.

Sample size	For in vitro experiments, 3 to 5 samples were used to to assess a biological effect. Most authors consider that replicating three times the same experiment, taking care that cell culture conditions do not change is enough to accurately describe a biological phenomenon. We increased the number of samples for qPCR experiments, where variability is higher. For in vivo experiments, each group of animals is composed of 8-10 individuals.
Data exclusions	No exclusion
Replication	The experiments are routinely conducted on several models in the lab.
Randomization	Random
Blinding	The experiments do not require blinding (only two main groups of tumors)

Reporting for specific materials, systems and methods

We require information from authors about some types of materials, experimental systems and methods used in many studies. Here, indicate whether each material, system or method listed is relevant to your study. If you are not sure if a list item applies to your research, read the appropriate section before selecting a response.

Materials & experimental systems

n/a	Involved in the study	
☐	☒	Antibodies
☐	☒	Eukaryotic cell lines
☒	☐	Palaeontology
☐	☒	Animals and other organisms
☒	☐	Human research participants
☒	☐	Clinical data

Methods

n/a	Involved in the study	
☒	☐	ChIP-seq
☐	☒	Flow cytometry
☒	☐	MRI-based neuroimaging

Antibodies

Antibodies used	ARD1: Dilution:1/2,000; Homemade - CD133: Dilution: 1/1,000; CST - CD146 (PE-conjugated): Dilution:1/1,000; BioCytex - CDH1: Dilution: 1/100; BD - CDH2; Dilution: 1/1,000; CST - CLDN1: Dilution: 1/100; CST - ERK (p42/44 MAPK); Dilution: 1/1,000; CST - Goat anti-rabbit IgG Alexa 488: Dil 1/500; Abcam - Goat anti-mouse IgG Alexa 594: Dil 1/500; Abcam - HRP-anti mouse: Dil 1/5,000; Promega - HRP-anti rabbit: Dil 1/5,000; Promega - p-ERK (pp42/44 MAPK): Dil 1/1,000; Abcam - PDPN: Dil1/25; Diagomics - Alpha-Tubulin: Dil 1/5,000; Invitrogen - VEGFC: Dil 1/100; R&D Systems.
Validation	All the commercial antibodies have been validated by the suppliers. All the homemade antibodies have been validated in the lab.

Eukaryotic cell lines

Policy information about cell lines

Cell line source(s)	ATCC - DSMZ
Authentication	The cell lines have been authenticated by the suppliers.
Mycoplasma contamination	All the cell lines were tested negative for mycoplasma in our hands.
Commonly misidentified lines (See ICLAC register)	None

Animals and other organisms

Policy information about studies involving animals; ARRIVE guidelines recommended for reporting animal research

Laboratory animals	We used Female Nude mice (NMRI-Foxn1nu/Foxn1nu) of 6 week old, from Janvier lab

Wild animals	None
Field-collected samples	None
Ethics oversight	Comité Institutionnel d'Éthique Pour l'Animal de Laboratoire (Institutional Ethics Committee for the Laboratory Animal), under the supervision of the Department of Advanced Education and Research

Note that full information on the approval of the study protocol must also be provided in the manuscript.

Flow Cytometry

Plots

Confirm that:

- [x] The axis labels state the marker and fluorochrome used (e.g. CD4-FITC).
- [x] The axis scales are clearly visible. Include numbers along axes only for bottom left plot of group (a 'group' is an analysis of identical markers).
- [x] All plots are contour plots with outliers or pseudocolor plots.
- [x] A numerical value for number of cells or percentage (with statistics) is provided.

Methodology

Sample preparation	Wild type or irradiation resistant Daoy or HD MB03 cells (106 cells) were softly dissociated with 1X accutase (HyClone HyQTase, Fisher Scientific). Accutase effects were stopped by resuspension of the cells in 1X PBS and centrifugation (250xg; 5min). Cells were counted (Coulter counter) and 1 million cells were washed in PBS-BSA0.5% for each condition. They were centrifuged again, in the same conditions as above and resuspended in 100 µl of PBS-BSA 0.5%.The cells were then labeled with 20 µl of a mouse monoclonal PE-conjugated anti-CD146, (BioCytex, Marseille, FRANCE), for 1h at 4°C. The cells were centrifuged and washed in PBS-BSA 0.5%, twice (as described above), and finally resuspended in 300 µl PBS-BSA 0.5% for flow cytometry measurements.
Instrument	BD BioSciences Facs CANTO
Software	Data were collected with the BD FACSDiva software application and analyzed on FlowJo
Cell population abundance	Abundance of the relevant cell population (single cells) is 15,000 - 20,000 cells
Gating strategy	In order to focus on our population of interest we used Forward and Side scatter gating strategy. We thus removed debris and groups of cells, and focused on the single cell population.

- [x] Tick this box to confirm that a figure exemplifying the gating strategy is provided in the Supplementary Information.
