# Supplementary material for: Author Correction: VEGFC negatively regulates the growth and aggressiveness of medulloblastoma cells
Source: Commun Biol. 2020 Dec 7;3:758. doi: 10.1038/s42003-020-01502-2 (PMC7721738; doi:10.1038/s42003-020-01502-2)
Supplement: Supplementary file 1 — Supplementary items [file 42003_2020_1502_MOESM1_ESM.pdf]

Supplementary figures

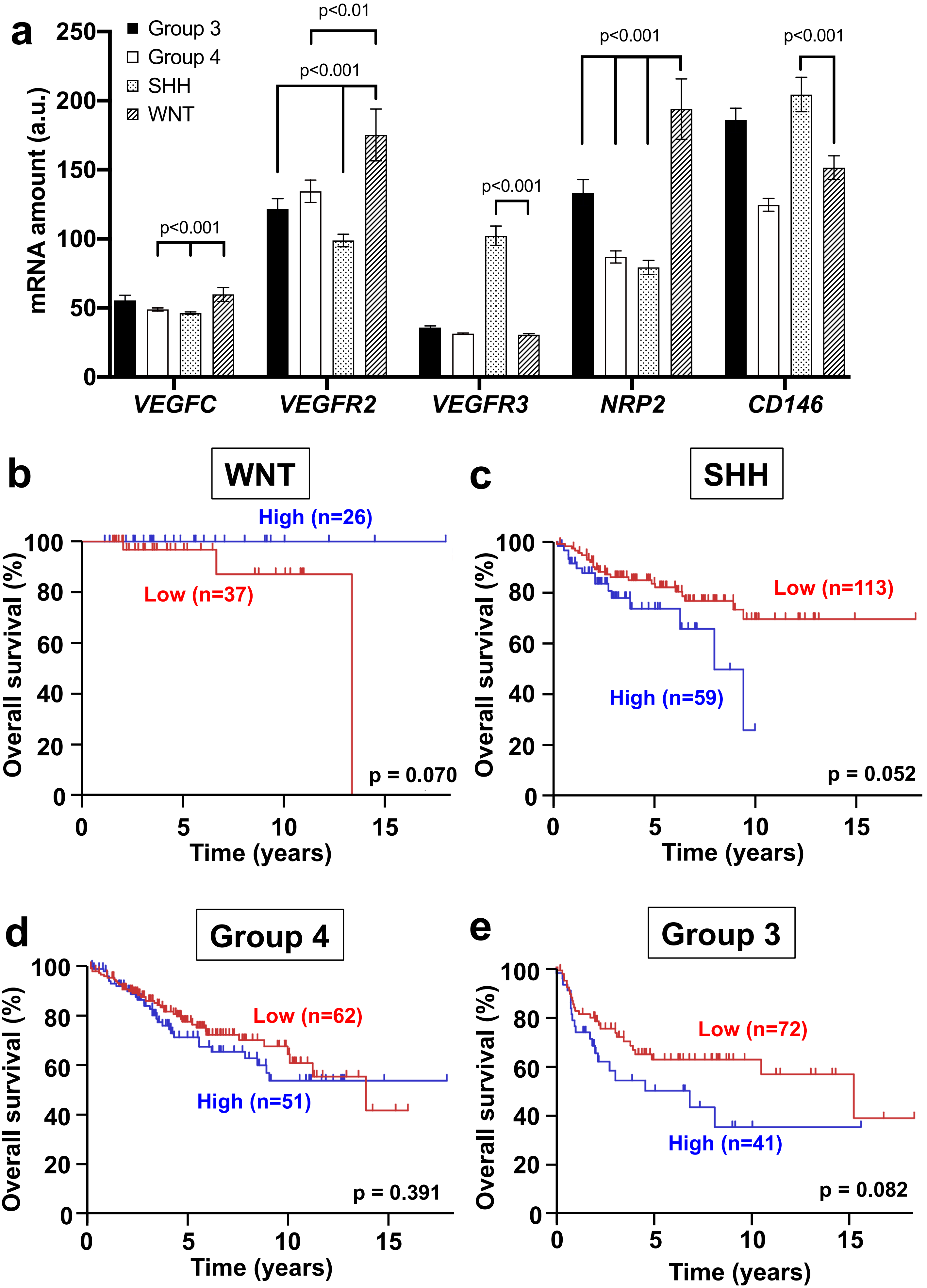

Supplementary Figure S1: Penco-Campillo *et al*

Supplementary Figure S1. Expression of VEGFC and its receptors in the different groups of MB. Correlation with MB aggressiveness. a. The amounts of VEGFC, VEGFR2, VEGFR3, NRP2 and CD146 genes were determined by analysis of the R2: Genomics Analysis and Visualization Platform (http://r2.amc.nl) data. Expression of one gene within the WNT subgroup was compared with expression of the same gene within every other subgroup (Multiple t-test comparison analysis). **b-e.** Overall survival of patients as a function of *VEGFC* expression was studied in the four MB subgroups, from the R2 Platform data. Cut-off: 40% mRNA expression. **b.** WNT subgroup. **c.** SHH subgroup. **d.** Group 4. **e.** Group 3.

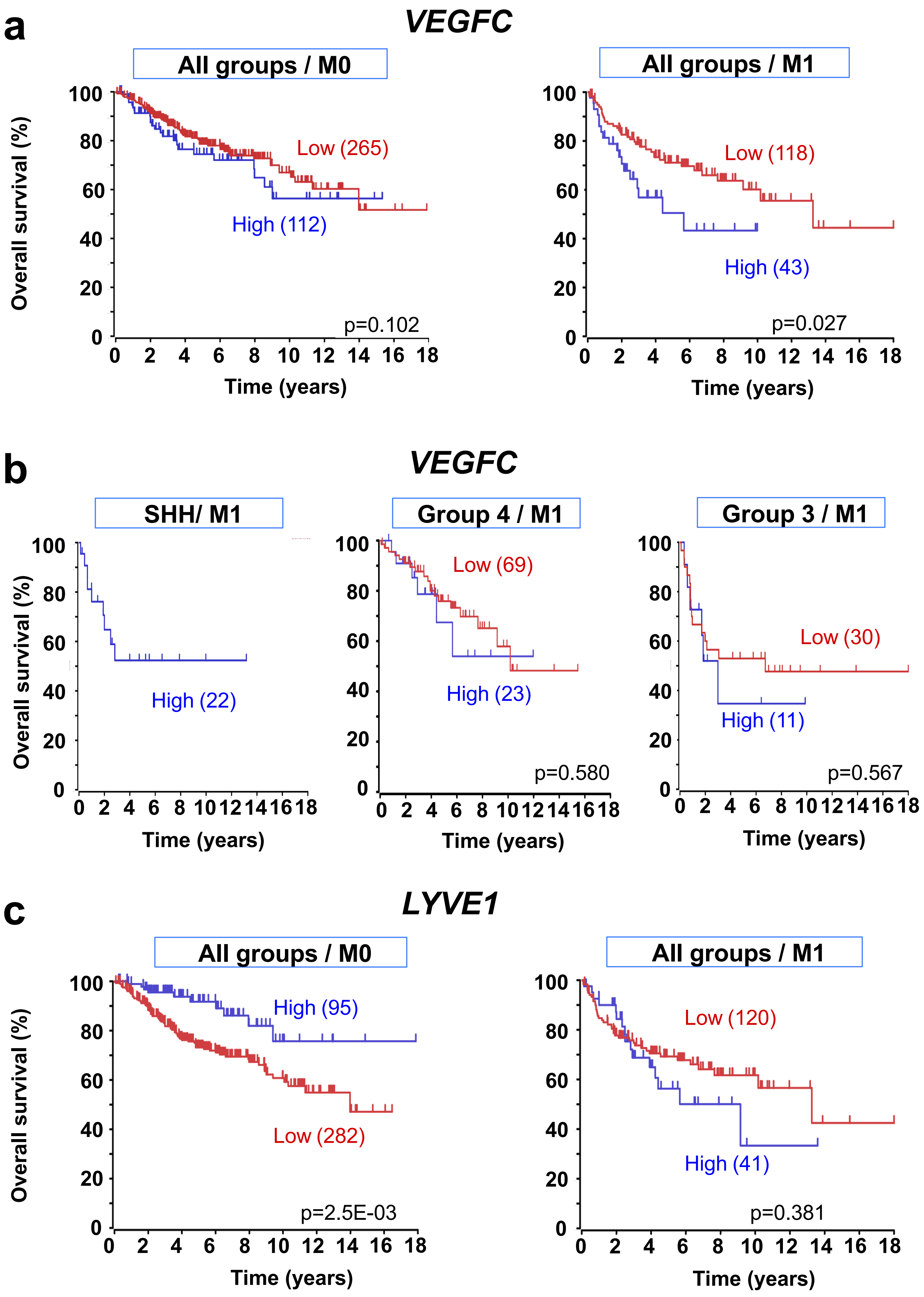

Supplementary Figure S2: Penco-Campillo *et al*

Supplementary Figure S2. Correlation between VEGFC/LYVE1 expression and MB aggressiveness. a. Overall survival of patients was determined by analysis of the R2: Genomics Analysis and Visualization Platform (http://r2.amc.nl) data as a function of VEGFC expression (One-way ANOVA and t-test comparison analysis). Data from all four groups of MB were analyzed as a pool (All groups) and the effect of metastatic status was analyzed (M0: non-metastatic; M1: metastatic). **b.** Overall survival of patients as a function of VEGFC expression was studied in the metastatic M1 group in the SHH sugroup, Group 4, Group 3. **c.** Overall survival of patients was determined as a function of LYVE1 expression in the "All groups" situation. Data were analyzed as a function of metastatic status. For all the graphs of Supp. Fig. S2, data were binned by 75% quartile.

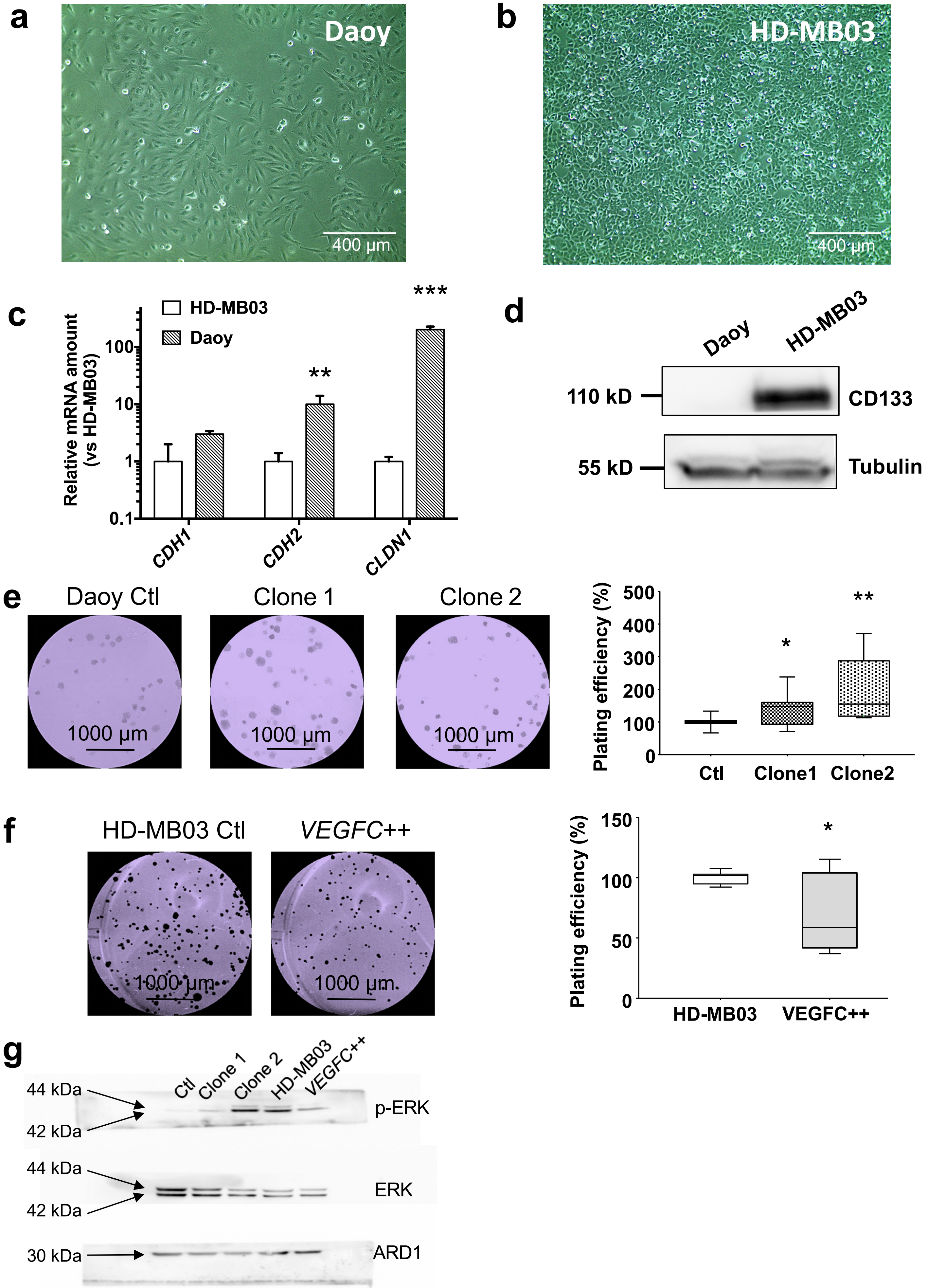

Supplementary Figure S3: Penco-Campillo *et al*

Supplementary Figure S3. Characterization of the phenotype of MB cells. a. Representative image of Daoy cells. **b.** Representative image of HD-MB03 cells. **c.** Relative expression of EMT gene mRNA in Daoy and HD-MB03 cells (n=3; **: p<0.01; ***: p<0.001). **d.** Representative immunoblot of CD133 showing that Daoy cells are CD133- and HD-MB03 are CD133+ cells. **e.** Representative experiment and quantification graph comparing the clonogenicity abilities of Daoy cells and Daoy *VEGFC*KO cells (n=4 independent experiments, each conducted in triplicates; *: p<0.05; **: p<0.01). **f.** Representative experiment and quantification graph comparing the clonogenicity abilities of HD-MB03 cells and *VEGFC*-overexpressing HD-MB03 cells (n=4 independent experiments, each conducted in triplicates; *: p<0.05). **g.** Representative immunoblot (uncropped, unprocessed membrane) showing the involvement of the ERK pathway in the VEGFC-dependent cell proliferation.

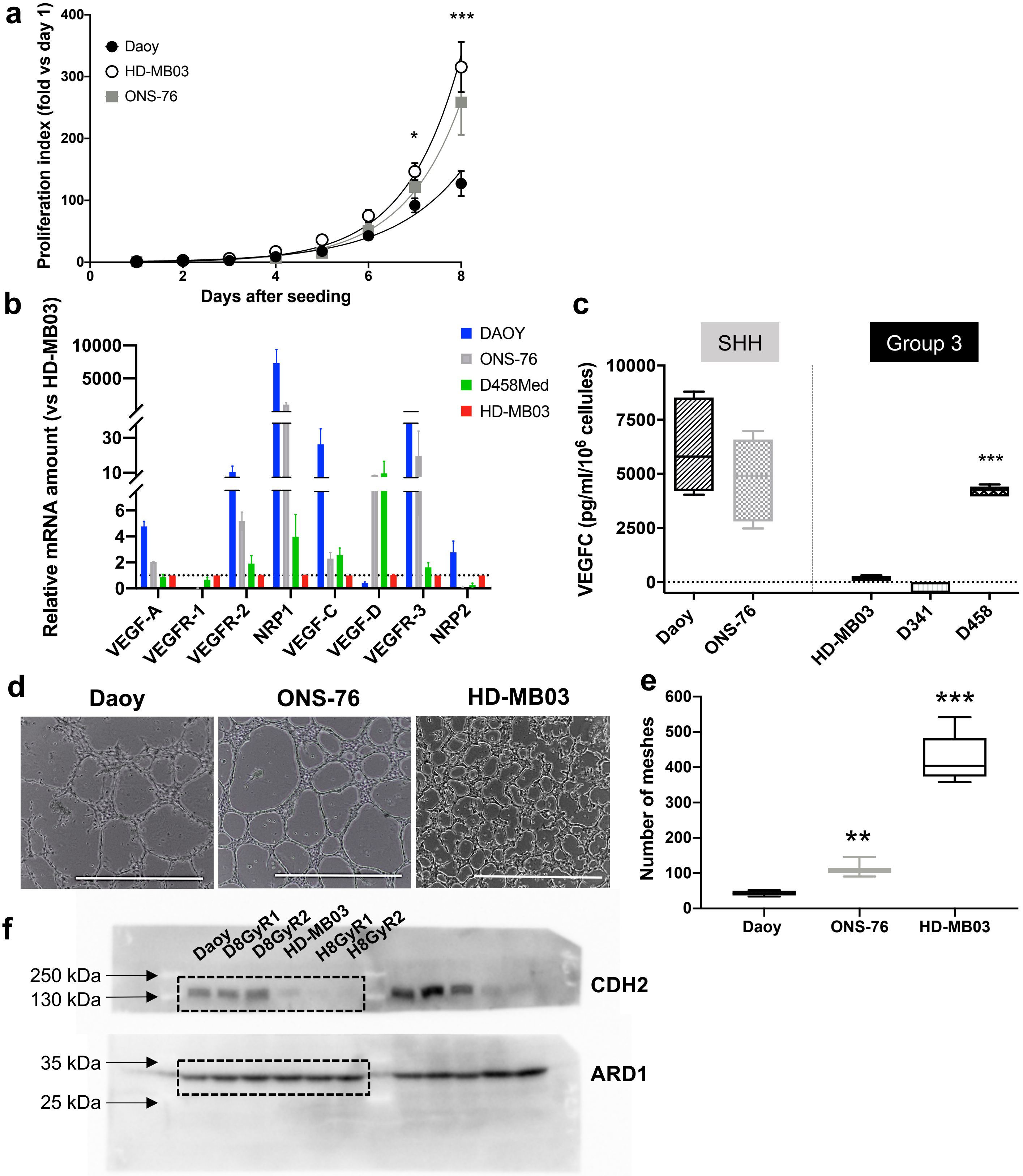

Supplementary Figure S4. MB group-related features of MB cells. a. Average Daoy, ONS-76 and HD-MB03 cell proliferation. Cell proliferation has been measured every day, for eight days. Proliferation of ONS-76 cells was compared to proliferation of Daoy cells. n=3 experiments; *: p<0.05; ***: p<0.001. **b.** Basal relative amount of VEGFC mRNA and lymphangiogenic related genes in two SHH-derived cell lines (Daoy, ONS-76) and two Group 3-derived MB cell lines (HD-MB03, D458Med), as measured by RT-qPCR. n=4 experiments; ANOVA; ***: p<0.001. **c.** Basal amount of VEGFC secreted by different MB model cells of the SHH subgroup and Group 3. n=3 experiments; p<0.001. **d.** Representative experiment showing the pseudo-tube formation in Daoy, ONS-76 or HD-MB03 cells; scale bar = 1000 μm. **e.** Quantification of the pseudo-tubes formed by each type of cells: average results (n=4); ***: p<0.001. **f.** Representative immunoblot (uncropped, unprocessed membrane) showing CDH2 expression in irradiation-resistant cells. ARD1 was used as loading control. Dashed lines enclose the zones shown in Fig. 5.

Supplementary Figure S4: Penco-Campillo *et al*

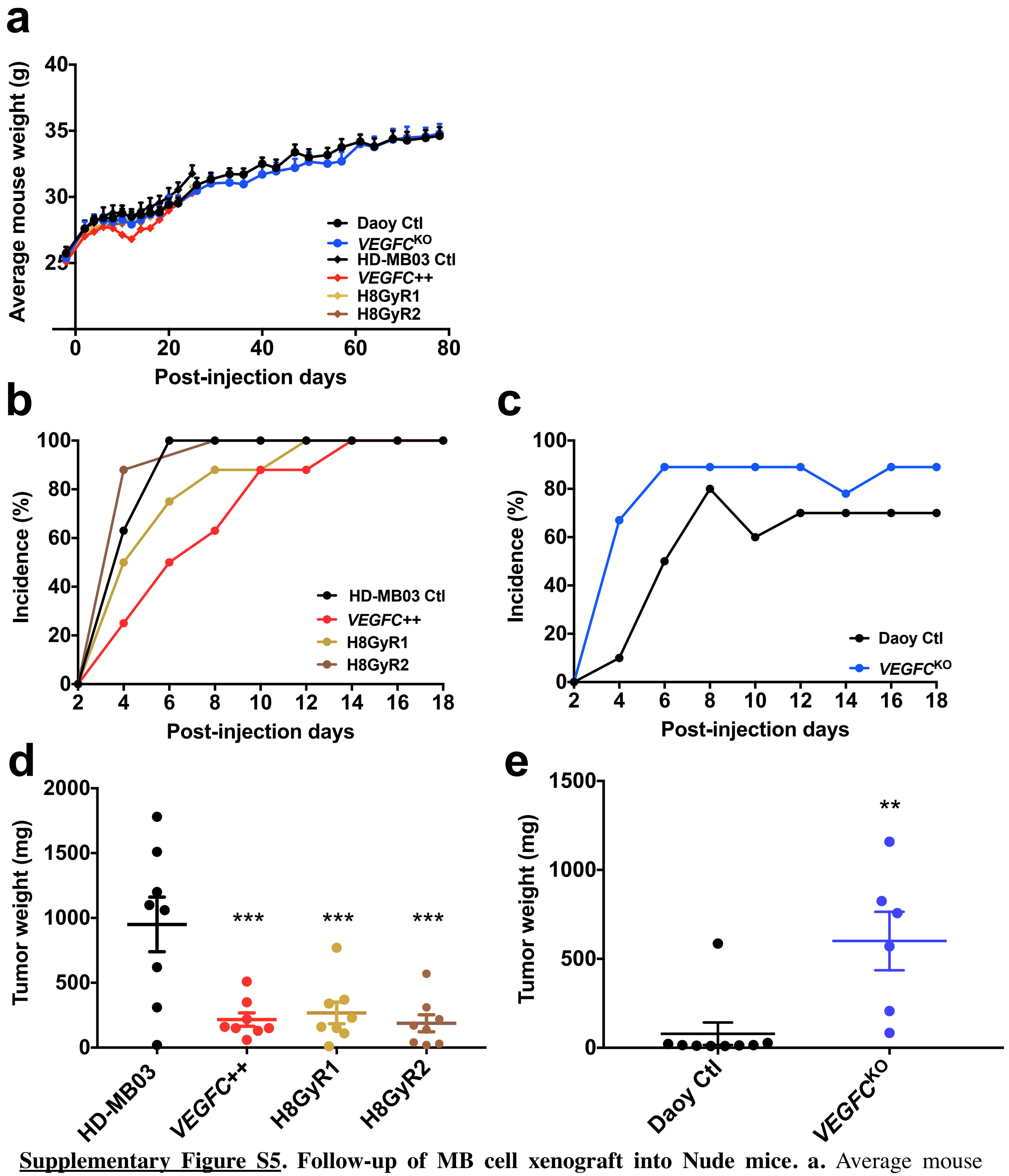

Supplementary Figure S5. Follow-up of MB cell xenograft into Nude mice. a. Average mouse weight was calculated every other day, all along the experiment (n=8 mice in each group). **b.** Tumor incidence (number of mice presenting a measurable tumor) was measured in each HD-MB03 derived cell-injected group. Measurements were stopped when the incidence curve plateaued (n=8 mice in each group). **c.** Tumor incidence was measured in each Daoy derived cell-injected group. Measurements were stopped when the incidence curve plateaued (n=8 mice in each group). **d.** HD-MB03-derived tumor weight at time of sacrifice. When the tumors reached 1000 mm^3, the animals (8 per group) were sacrificed and the tumors harvested. They were immediately weighed and processed for analysis. **e.** Daoy-derived tumor weight at time of sacrifice. When the tumors reached 1000 mm^3, the animals (6 or 9 per group) were sacrificed and the tumors harvested. They were immediately weighed and processed for analysis.

Supplementary Figure S5: Penco-Campillo *et al*

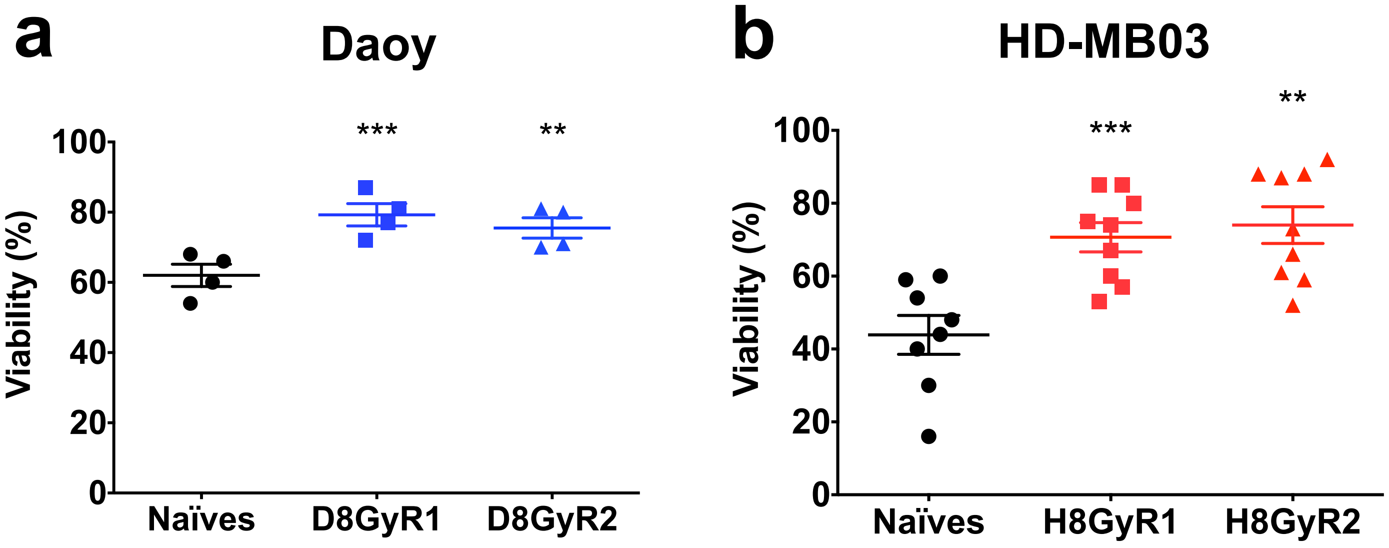

Supplementary Figure S6. Viability of MB cells after 10 cycles of X-Ray irradiation (8 Gy). Two populations of naïve Daoy (**a**) or HD-MB03 (**b**) cells have been irradiated weekly, for 10 weeks. An eleventh irradiation was conducted to perform viability tests. Daoy cell survival was 80% vs. 60% for naïve cells. HD-MB03 cells survived at 70% vs. 50% for naïve cells. As such, the cells were considered resistant to irradiation.

a

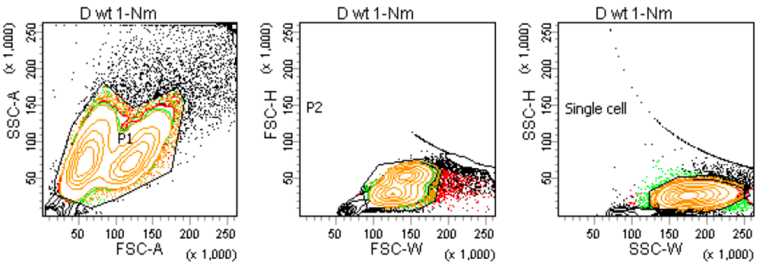

b

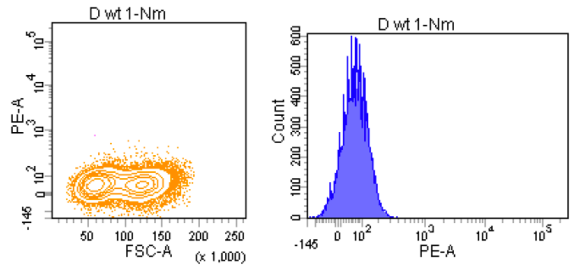

c

Tube: Nm

Population	#Events	%Parent	%Total
All Events	22,736	####	100.0
P1	15,982	70.3	70.3
P2	15,551	97.3	68.4
Single cell	14,890	95.7	65.5
PE+	1	0.0	0.0

Tube Name: Nm

Population	#Events	%Parent	PE-A Mean
All Events	22,736	####	81
P1	15,982	70.3	64
P2	15,551	97.3	63
Single cell	14,890	95.7	62
PE+	1	0.0	788

d

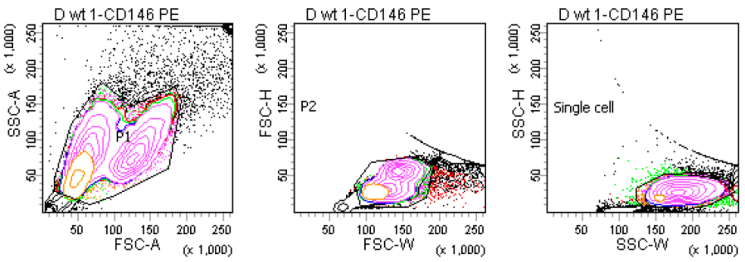

e

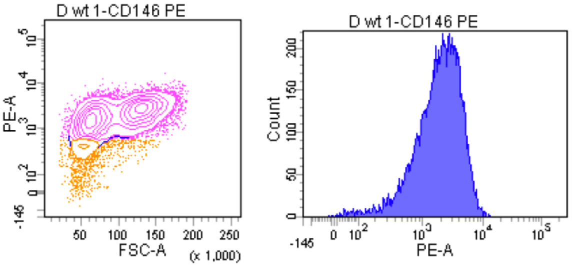

f

Tube: CD146 PE

Population	#Events	%Parent	%Total
All Events	14,152	####	100.0
P1	11,091	78.4	78.4
P2	10,855	97.9	76.7
Single cell	10,326	95.1	73.0
PE+	9,114	88.3	64.4

Tube Name: CD146 PE

Population	#Events	%Parent	PE-A Mean
All Events	14,152	####	2,731
P1	11,091	78.4	2,221
P2	10,855	97.9	2,215
Single cell	10,326	95.1	2,219
PE+	9,114	88.3	2,467

Supplementary Figure S7. Gating strategy used to measure the expression of CD146 at the plasma membrane of MB model cells (Daoy cells as an example). a. Gating strategy (unlabeled cells). **b.** Visualization of unlabeled cells. **c.** Cell population arborescence table generated from unlabeled cells. **d.** Gating strategy (labeled cells). **e.** Visualization of P-E labeled cells (expressing CD146). **f.** CD146-labeled cell population arborescence table.

Supplementary Figure S7: Penco-Campillo *et al*

Supplementary tables

Supplementary Table S1. Correlation between the expression of mRNAs of the VEGFC / VEGFC receptor axis and MB aggressiveness. The amounts of *VEGFC, VEGFR3, NRP2,* and *CD146* genes were determined by analysis of the R2: Genomics Analysis and Visualization Platform (http://r2.amc.nl) data. Data are mean ± S.E of n = number of patients. Two-way ANOVA; p<0.001.

SUBGROUP	*VEGFC*	*VEGFR3*	*NRP2*	*CD146*	NUMBER OF PATIENTS
**GROUP 3**	55.271 ± 3.878	35.826 ± 1.150	133.381 ± 9.506	185.733 ± 8.840	144
**GROUP 4**	49.746 ± 1.029	31.889 ± 0.554	87.135 ± 3.866	124.897 ± 4.212	326
**SHH**	46.230 ± 3.878	102.200 ± 1.152	79.347 ± 9.532	204.469 ± 8.883	223
**WNT**	59.793 ± 5.082	30.646 ± 0.797	193.894 ± 22.022	151.477 ± 8.656	70

Supplementary Table S2. VEGFC invalidation by CRISPR-Cas9. Wild-type, fake-mutated (Ctl) and mutated sequences. *: Stop codon.

Clone	Nucleotide/protein sequence
***VEGFC* WT**	CAGTTACGGTCTGTGTCCAGTGTAGATGAACTCATGACTGTA<br>Q L R S V S S V D E L M T V
***VEGFC* Ctl**	CAGTTACGGTCTGTGTCCAGTGTAGATGAACTCATGACTGTA<br>Q L R S V S S V D E L M T V
***VEGFC*KO Clone 1**	CAGTTACGGTCTGTGTCCAGTGT----GAACTCATGACTGTA<br>Q L R S V S S V N S *
***VEGFC*KO Clone 2 (Allele 1)**	CAGTTACGGTCTGTGTCCAGT-TAGATGAACTCATGACTGTA<br>Q L R S V S S *
***VEGFC*KO Clone 2 (Allele 2)**	CAGTTACGGTCTGTGT--A--GTAGATGAACTCATGACTGTA<br>Q L R S V *

Supplementary Table S3. List of antibodies used in this study.

Antibody	Experiment	Dilution	Supplier	Reference #
**ARD1**	Immunoblotting	1/2,000	**Homemade**	**N/A**
**CD133**	Immunoblotting	1/1,000	**CST**	**D2V8Q**
**CD146 (PE-conjugated)**	Flow cytometry	1/1,000	**BioCytex**	**#17327**
**CDH1**	Immunocytochemistry	1/100	**BD**	**#610181**
**CDH2**	Immunoblotting	1/1,000	**CST**	**#9272**
**CLDN1**	Immunocytochemistry	1/100	**CST**	**D5H1D**
**ERK (p42/44 MAPK)**	Immunoblotting	1/1,000	**CST**	**#4695S**
**Goat anti-rabbit IgG Alexa 488**	Immunocytochemistry	1/500	**Abcam**	**ab150077**
**Goat anti-mouse IgG Alexa 594**	Immunocytochemistry	1/500	**Abcam**	**ab150120**
**HRP-anti mouse**	Immunoblotting	1/5,000	**Promega**	**W4021**
**HRP-anti rabbit**	Immunoblotting	1/5,000	**Promega**	**W4011**
**p-ERK (pp42/44 MAPK)**	Immunoblotting	1/1,000	**Abcam**	**ab32538**
**PDPN**	Immunohistochemistry	1/25	**Diagomics**	**BSB6067**
**a-Tubulin**	Immunoblotting	1/5,000	**Invitrogen**	**A11126**
**VEGFC**	Immunocytochemistry	1/100	**R&D Systems**	**AF752**
